# Ex vivo single‐cell profiling of acute myocardial infarction patients reveals disproportionate CD66b ^+^ cell secretion response

**DOI:** 10.1002/btm2.70043

**Published:** 2025-07-07

**Authors:** Kerwin Kwek Zeming, Ri Lu, Elizabeth Lee, Ka‐Wai Cheung, Nicholas W. S. Chew, Kai Lee Woo, Lih Feng Cheow, Jongyoon Han, Shir Lynn Lim

**Affiliations:** ^1^ Critical Analytics for Manufacturing of Personalised Medicine Singapore‐MIT Alliance for Research and Technology Singapore Singapore; ^2^ Graduate School for Integrative Sciences and Engineering National University of Singapore Singapore Singapore; ^3^ Department of Cardiology National University Heart Centre Singapore Singapore; ^4^ Department of Biomedical Engineering National University of Singapore Singapore Singapore; ^5^ Department of Electrical Engineering and Computer Science Massachusetts Institute of Technology Cambridge Massachusets USA; ^6^ Department of Biological Engineering Massachusetts Institute of Technology Cambridge Massachusets USA; ^7^ Yong Loo Lin School of Medicine National University of Singapore Singapore Singapore; ^8^ Pre‐hospital Emergency Research Centre, Health Services and Systems Research Duke‐NUS Medical School Singapore Singapore

**Keywords:** acute myocardial infarction, deterministic lateral displacement, droplet microfluidics, immune profiling, Secretomics, single cell

## Abstract

Acute myocardial infarction (AMI), a leading cause of death globally, triggers complex inflammatory responses critical to patient outcomes. However, rapid tools for profiling immune responses at the single‐cell level are lacking. The integrated Single‐cell Enzyme and Antigen Quantification (iSEAQ) system addresses this gap by enabling high‐throughput, single‐cell analysis of immune cell activity using just 20 μL of blood. This novel tool processes live CD66b and CD3 cells to quantify the secretion of Granzyme B, Neutrophil Elastase, and CD31 within minutes. Longitudinal studies on nine AMI patients revealed that CD66b^+^ cells are major contributors (up to 95%) to key inflammatory enzymes, including the unexpected secretion of Granzyme B. iSEAQ achieves unparalleled sensitivity (0.4 fg/cell) and predictive accuracy (>90%) for patient profiling across AMI onset, treatment, and discharge. This innovation provides clinicians with a rapid, precise method to monitor immune responses, unveiling new insights into AMI inflammation and therapy.

**Translational Impact Statement**

iSEAQ platform delivers high‐sensitivity (0.4 fg/cell), single‐cell analysis of immune secretion within 30 min using a drop of blood, uncovering that CD66b^+^ granulocytes dominate inflammatory enzyme release in AMI, particularly Granzyme B secretion. Using this secretion data, we developed an immune response model correlating with the AMI patient journey from admission, treatment, to discharge. This can potentially inform treatment strategies and reduce post‐infarction complications. Beyond AMI, iSEAQ can guide personalized anti‐inflammatory interventions and has broad potential for other inflammatory diseases, facilitating faster, tailored therapeutic decisions.

## 1 INTRODUCTION

Acute myocardial infarction (AMI), a leading cause of mortality and morbidity worldwide,^1^, ^2^ triggers a complex and dynamic inflammatory cascade that plays a critical role in determining the clinical outcome. This cascade involves an orchestrated response from various cellular components of the cardiac and immune systems, influencing myocardial infarct size, adverse left ventricular (LV) remodeling, and patient recovery.^3^, ^4^, ^5^, ^6^ The inflammatory response post‐AMI is not merely a pathological process; rather, it embodies a double‐edged sword, essential for tissue repair and regeneration but also capable of exacerbating injury when dysregulated.^7^ Understanding this intricate response provides valuable insights into potential therapeutic interventions that aim to improve AMI outcomes.

Central to the inflammatory response in AMI is leukocytosis,^8^ characterized by the activation and dynamic interaction of various leukocyte sub‐populations.^9^, ^10^ Neutrophils are among the first responders in AMI,^11^ which undergo a significant transformation from a pro‐inflammatory state immediately post‐injury to an anti‐inflammatory state during the reparative phase.^12^, ^13^ As the post‐AMI immune landscape evolves, other immune cells such as monocytes,^14^ macrophages,^15^ B cells,^16^ and T lymphocytes^17^ also contribute, each in a unique way. Clinically detecting these evolving dynamic transitions remains challenging and impossible at point‐of‐care, a task complicated by the limitations of current research methodologies, particularly in live single‐cell secretion assays.

Current research into AMI's cellular mechanisms also faces significant limitations due to the reliance on animal models, such as murine^13^, ^18^ and porcine^19^, ^20^ studies, as well as in vitro systems that may not accurately replicate human conditions.^21^, ^22^ For instance, the bulk of immune response research in AMI stems from immunohistology of cardiac tissues in animal models^23^, ^24^ and this does not capture pre‐intravasation immune activities and the human anatomy, leading to potential misinterpretations in human AMI.^25^ Furthermore, many single‐cell gene sequencing studies focus on peripheral mononuclear fractions, overlooking critical contributions of the diverse polynuclear cells like granulocytes, crucial in cardiac repair and AMI inflammatory response.^26^ While various single‐cell profiling techniques can yield useful information on gene transcription and cell pathways, the process requires substantial resources, extensive sample preparation and analysis, often extending more than a week.^27^ This delay presents a substantial challenge in AMI, a rapidly evolving medical condition where timely intervention is crucial.^28^ A tool enabling rapid, physiologically relevant profiling of single‐cell immune responses would be a significant advancement in AMI research, offering immediate and actionable clinical insights that current methodologies cannot provide.

To address these gaps, we introduce the ex vivo integrated Single‐cell Enzyme and Antigen Quantification (iSEAQ) system, a pioneering tool in cardiac immunology. iSEAQ, capable of high‐throughput, sensitive, quantitative single‐cell assays, probes leukocytes for secreted enzymes of neutrophil elastase (NE), granzyme B (GZB), and surface antigens (CD66b, CD3, and CD31) within 30 min. This ex vivo profiling system employs a closed‐loop, pressure‐driven microfluidic process for efficient processing of whole blood, achieving a throughput of 10–20 million blood cells per minute. iSEAQ's sensitivity enables detection of biomarkers as low as 0.4 fg per cell, offering new insights into the immune response dynamics in AMI within the first 24 h post‐admission.

Our study focuses on the unique functional secretion of enzymes by CD66b^+^ granulocytes in AMI patients, revealing their disproportionate role in the peripheral blood inflammatory response, and underscoring their significant contribution to AMI inflammation. This discovery has profound implications for AMI diagnosis and treatment, pinpointing specific granulocyte subsets as potential therapeutic targets. The iSEAQ profile's ability to define these subsets could revolutionize AMI clinical management, offering more precise strategies to mitigate the adverse effects of inflammation. This study marks a significant advancement in cardiac immunology, highlighting the pivotal role of CD66b^+^ cells in the inflammatory cascade and opening avenues for targeted therapies to improve AMI patient outcomes. The use of iSEAQ as a quantitative measure of active immune response of these markers at a single‐cell resolution and precision is unprecedented in the field of cardiac immunology, enabling the monitoring of functional immune responses in AMI with a temporal granularity that was previously unattainable.

## 2 MATERIALS AND METHODS

### 2.1 Study design and sample collection

This prospective observational study was conducted in the National University Hospital, Singapore. Patients were eligible if they were at least 21 years of age, presented with typical signs and symptoms of ST‐segment elevation myocardial infarction (STEMI) using internationally accepted criteria, and were able to provide written consent. We excluded patients who had a hemoglobin level <10 g/dL, were terminally ill with an expected life expectancy of <6 months, or were pregnant women. All participants (patients and healthy controls) provided written informed consent, and this study was approved by the Institutional Review Board (DSRB 2021/00246).

Venous whole blood, approximately 2 mL each, was drawn from healthy donors and patients at 3 timepoints: within 4 h of admission for STEMI, at 24 h after admission, and pre‐discharge. These whole blood samples were collected in 3 mL ethylenediamine tetraacetic acid (EDTA) tubes, kept at room temperature, and processed within 1 h of collection.

### 2.2 Microfluidic fabrication

The iSEAQ microfluidic device is an integrated microfluidic chip based on deterministic lateral displacement (DLD) L‐shape pillars adapted from Zeming et al.^29^ with the use of L‐shape pillars in the microfluidic device (see Figure S1 and Table S1). The design specification uses a critical cut‐off of *D*_*c*_ of 5.5 μm^29^ (see Figure S1) for efficient isolation of immune cells from whole blood. The fabrication method is based on our previous study.^29^ Briefly, the microfluidic chips were made from polydimethylsiloxane (PDMS, Sylgard 184, Dow Corning) using soft‐lithography replica molding from a master SU‐8 coated 4‐inch silicon wafer master‐mold fabricated with standard photolithography methods using a quartz chromed mask (JDphotodata, UK). The cast PDMS is peeled off from the mold, diced, and holes are punched for the inlets and outlets using a 1.5 mm biopsy punch. The PDMS device is bonded onto a glass substrate using an air plasma surface treatment machine. The surface of the PDMS device is made hydrophobic using a chemical vapor deposition method for at least 2 h using trichloro(1H,1H,2H,2H‐perfluorooctyl) silane (Sigma, Singapore).

### 2.3 Integrated DLD microfluidics for iSEAQ

The microfluidic device using the L‐shape pillars is optimized for sorting of leukocytes based on the DLD specifications and sorting efficiency of >99%.^29^ This sorting efficiency of >95% for immune cells are also reported by various groups using DLD for immune cell isolation,^30^, ^31^, ^32^ albeit with varying DLD pillar shapes. Campos‐González et al. went a step further to evaluate immune cell activation post‐sorting in DLD devices and found that the activation was negligible even after 1‐h post‐sort.^30^ In iSEAQ, the leukocytes were simultaneously sorted and suspended in an enzyme FRET substrate medium to be encapsulated in water‐in‐oil droplets. The droplets were generated and incubated at room temperature for up to 30‐min, then reinjected into a standard cross junction for oil spacing between droplets to ensure consistent droplet spacing. Samples and buffers were contained into tube reservoirs which were subsequently injected into the device at a pressure of 450mBar to ensure effective sorting of immune cells from whole blood. Based on device characterization from our earlier study,^29^ the immune cells are sorted well at 450 mBar in the L‐shape device (Figure S2) with a throughput of approximately 3 μL/min (Figure S3). The device for cell encapsulation in droplets were characterized previously in Zeming et al. showing consistent droplet size generation and controlled droplet generation (Figure S3). The microfluidic designs and fluid balancing calculations are shown in Figure S4.

### 2.4 Experimental setup

**FIGURE 1:**
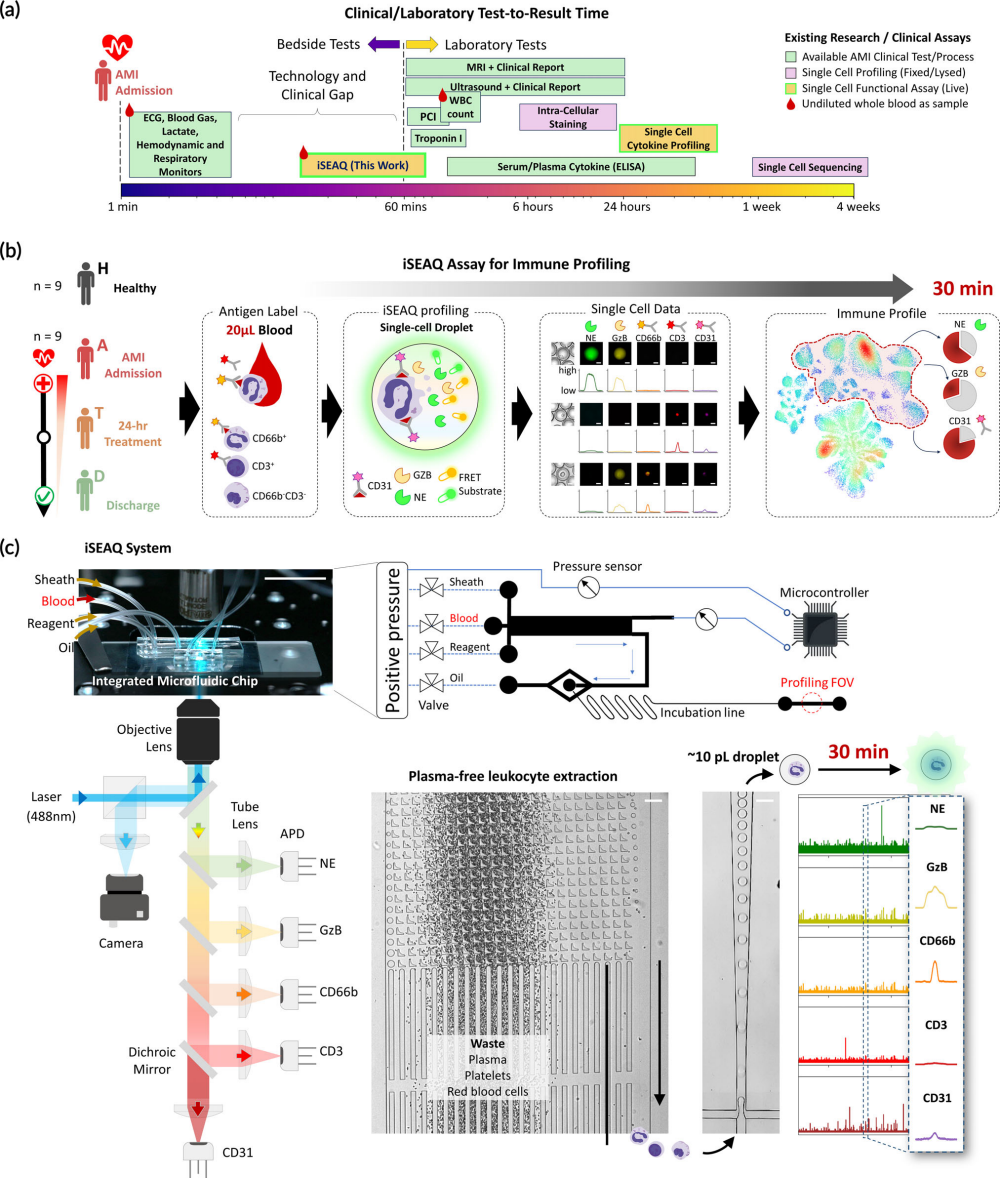
Schematics for integrated Secreted Enzyme and Antigen Quantification (iSEAQ) assay and study design. (a) Overview of the current state of the art in clinical assessment of AMI and advanced tools available for immune profiling. (b) Workflow of iSEAQ assay with four clinical cohorts studied, namely healthy (H), AMI admission (a), 24 h. treatment (T), and discharge (d). 20 μL of whole blood was taken from each participant in this study. Each blood sample was stained with CD marker labels and processed in a 30‐min assay to obtain a single‐cell leukocyte profile of NE, GZB, CD66b, CD3, and CD31. (b) System schematics for the iSEAQ instrument. Two subsystems were developed for iSEAQ. (1) An integrated microfluidics chip driven by a closed‐loop pressure, which achieves leukocyte extraction, single‐cell droplet isolation, incubation, and profiling. (2) A 5‐channel fluorescence sampling platform for high‐throughput droplet signal 18‐bit readout. A camera was built into the system to provide image for laser alignment to profiling field‐of‐view (FOV) of the microfluidic device (Figure S5). The arrows indicate the leukocyte flow direction. Scale bars are 50 μm. APD, avalanche photodiode.

The device was primed via the inlets using a positive pressure of 450 mBar with a 0.2% (w/v) Pluronic F127 solution (Sigma, Singapore). Picosurf 5% (Spherefluidics, UK) surfactant was added to the Novec 3 M 7500 oil at a 2% v/v ratio and infused into the oil inlet (Figure 1). With the primed device, a 4‐valve luer manifold (Qosina, USA) was connected to four fluid reservoirs made from custom 1.5 mL Eppendorf tubes, which in turn were linked to the inputs of the device. To run iSEAQ, the reservoirs were pressurized to 450 mBar to initiate the fluid flow.

### 2.5 Optical and detectors

iSEAQ uses a custom system setup built from scratch with a free‐space continuous wave 488 nm blue laser (Coherent Sapphire LP) as the excitation light, as shown in Figure S5. The laser excites the fluorophores through a 20× 0.7NA Olympus objective, which also provides the illumination for the CMOS camera (Figure S1b, FLIR Blackfly S, US). The light is back propagated into the objective and through mirrors, and split into sequential low‐pass filters at wavelengths 525 nm, 580 nm, 630 nm, 690 nm, and a high‐pass filter at 780 nm (Figure S5). The corresponding fluorescence outputs were detected using Hamamatsu C12703 avalanche photodiodes (APD). The voltage signals were converted in real time using 18bit DAQ (USB‐1808X) for downstream data processing. Cell detection was performed using a photodiode (Thorlabs) to measure light blocked by passing cells.

### 2.6 Enzyme FRET substrates and fluorophore

NE substrate 5FAM‐TSFIRWTG‐K(CPQ2)‐NH2 (CPC Scientific) was designed based on a specific NE target peptide reported by Schulenburg et al.^33^ GZB substrate TQ3‐VGPDFGR‐mfluor blue 570 (AAT Bioquest) was designed based on a specific GZB peptide target validated by Choi et al.^34^ and applied by Kula et al.^35^ and Chiusolo et al.^36^ Immune cell surface markers were selected, respectively, CD66b‐PE dazzle (Biolegend, cat. 305122), CD3‐PE cy5.5 (Invitrogen, cat. 35‐0036‐42), and CD31‐PE cy7 (Biolegend, cat. 303118). Calibration of the iSEAQ was based on the corresponding substrates and CD markers.

### 2.7 Chemicals and buffers

Picosurf 5% (Spherefluidics, UK) surfactant was added to Novec 7500 oil at a 5% v/v ratio to be used for water‐in‐oil droplet generation. 0.2% Pluronic F127 (Sigma, SG) was diluted in 1× PBS (Sigma, SG) and used as a priming buffer, and 1× PBS was used as a washing buffer. The substrate buffer was prepared by adding 3 μM NE substrate, 5 μM GZB substrate, and 1% BSA to 1× PBS.

### 2.8 Python data and statistical processing

All data was processed in Python using libraries Numpy, Pandas, Scipy, Scikit Learn, Seaborn, Matplotlib, and UMAP. The 5‐channel fluorescence data was imported and transformed using the compensation matrix. Droplet signals were detected using a moving median algorithm (Figure S6). Ten empty droplet measurements were pooled to compute local medians of each of the five channels as a reference background value (Discussion S1). Student's two‐tailed *t*‐tests were performed for all samples, with paired t‐tests for all AMI patients and unpaired t‐tests for healthy donors.

## 3 RESULTS

### 3.1 Study population and panel selection

AMI is a heterogeneous condition. Acute ST‐elevation myocardial infarction (STEMI) is a specific type of AMI that has the worst short‐term prognosis among all AMI variants.^37^ We recruited nine STEMI patients (Table S2) and 10 healthy controls (HC) for this study. Among the STEMI patients, four had anterior STEMI and five had inferior STEMI. All were discharged alive after a median stay of 2 days (see Section 2). To provide individualized longitudinal evaluation of the dynamic immunological landscape during the hospital admission of STEMI, we profiled each patient at three specific timepoints during the index admission: on admission (A) before receiving emergency coronary angiogram and revascularization through percutaneous coronary intervention (PCI), 24 h post‐AMI (T), and on day of discharge from hospital (D) (Figure 1). For ease of reading, AMI is used in‐lieu of STEMI.

### 3.2 Development and functionality of the iSEAQ system for rapid immune profiling

The iSEAQ system investigated here aims to address a technology and clinical gap where existing clinical tools for AMI evaluation are lacking in terms of rapid and quantifiable immune response (Figure 1a). Even non‐invasive methods such as magnetic resonance imaging (MRI) and ultrasound require 24‐h turn‐around time for the clinical analysis and report. Many existing single‐cell immune profiling techniques require more than a week for the results, limiting the use cases to chronic diseases or research use only. Thus, the iSEAQ system developed here is a fully customized droplet fluorescence cytometry setup developed in this study to measure up to five proteomic targets directly from whole blood samples in a 30‐min process (Figure 1b). Compared to the cell profiling technique developed earlier,^29^ the main significant enhancements in the technology of iSEAQ include increased sensitivity to detect surface fluorescence markers, resulting in shortened cell incubation times and label‐free FTL for the detection of cells within the droplet with real‐time 5‐channel data processing. Clinically, this iSEAQ panel probes inflammation with granularity over the immune subtype of AMI patients compared to the indiscriminate immune cell secretion profile in acute heart failure patients (see Table S1). The panel consists of two secreted enzymes (NE and GZB) and three surface antigens (CD31, CD66b and CD3) biomarkers (Figure 1b). The CD66b and CD3 were selected to identify four immune phenotypes, namely granulocytes (CD66b^+^CD3^−^), T lymphocytes (CD66b^−^CD3^+^), a rare subclass of granulocytes (CD66b^+^CD3^+^) and a collection of all remaining immune phenotypes (CD66b^−^CD3^−^) shown in Table S3.

The iSEAQ is initiated by adding the cocktail of surface antigen fluorescence labels (CD66b, CD3, CD31) into 20 μL of undiluted whole blood sample (Figure 1b). The sample is subsequently processed through the integrated microfluidic device which performs the necessary leukocyte extraction, washing, and single‐cell encapsulation with NE and GZB enzyme substrates (Figure 1c). This critical step, which involves extracting immune cells and capturing their response in droplets as natively as possible, is performed in a continuous flow manner.

### 3.3 Quantitative correlation of enzyme activity and antigen concentration

Visual examination of the heterogeneous expression of the iSEAQ panel (NE, GZB, CD66b, CD3, and CD31) on leukocytes was performed on fluorescence microscopy images of single‐cell droplets. Bright field (BF) images were used to capture leukocytes within droplets (Figure S7). Enzyme secretion measurements were detected by the overall droplet fluorescence when the secreted enzymes cleave the Fluorescence Resonance Energy Transfer (FRET) substrates within the droplet. The fluorescence levels of the pixels were integrated across the images resulting in the intensity plot, which was comparable to data extracted from the iSEAQ flow fluorescence signal.

**FIGURE 2:**
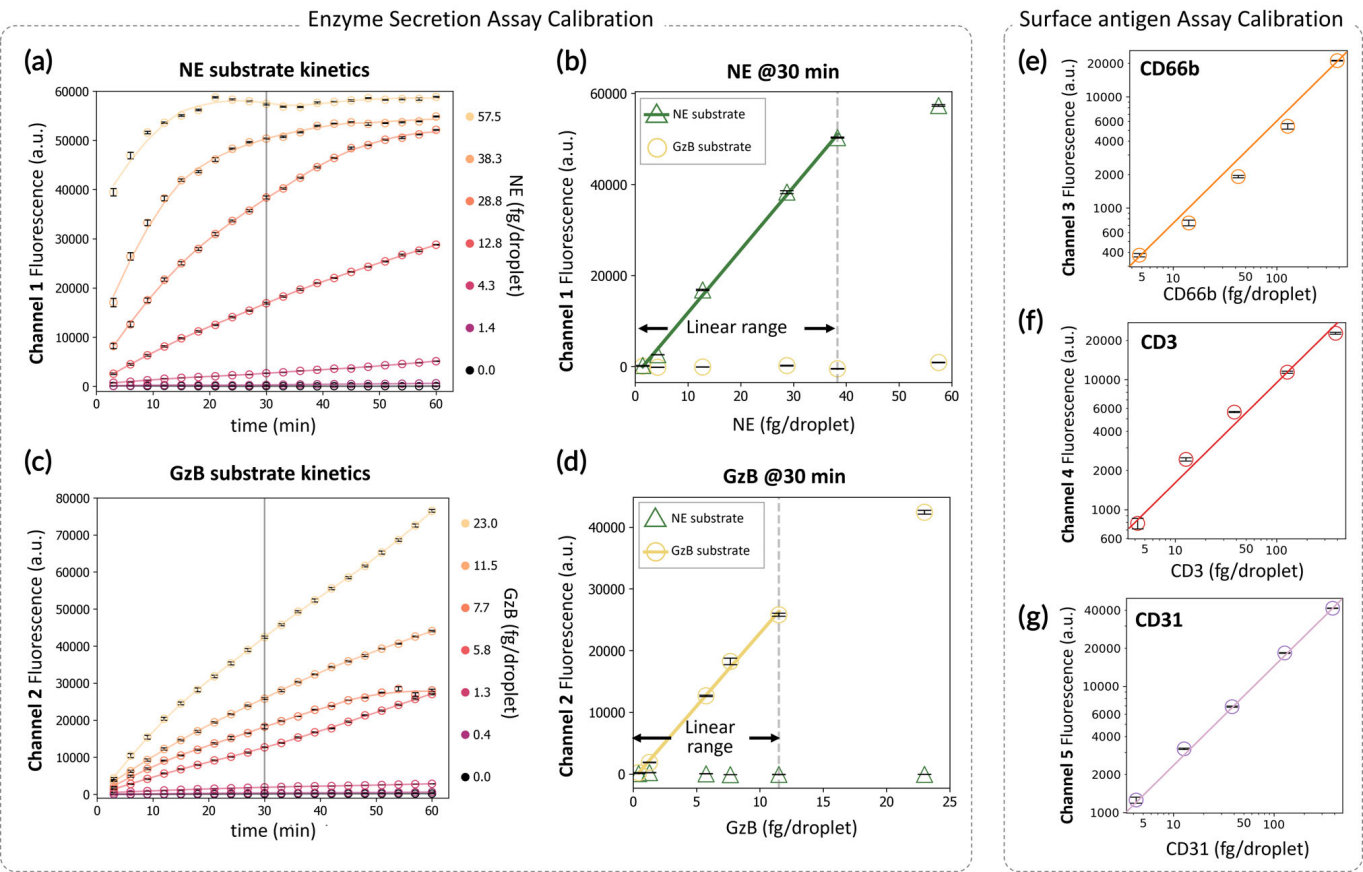
Characterizations and calibration of iSEAQ assay enzyme secretion and surface antigen markers. (a) shows the characterization of human neutrophil elastase (NE) FRET substrate with the kinetic curve of fluorescence intensity up to 60mins at varying enzyme concentrations. (b) shows the approximate linear range of the calibration curve at 30 min of droplet incubation. (c) shows the kinetics of granzyme B (GZB) FRET substrates with varying concentrations of GZB enzyme. (d) shows the corresponding calibration curve at 30 min showing the linear dynamic range of GZB enzyme detection. (e–g) Calibration curves of CD66b, CD3, and CD31.

Standard curves for all five iSEAQ proteomics signals were performed to establish quantitative measurement of enzyme and surface antigens. The amount of fluorescence intensity in NE and GZB channels for a given assay time corresponds to the kinetic activity and concentration of enzymes, which is calibrated in Figure 2a,c. Based on the enzyme kinetics, 30 min was selected as the ideal reaction time to balance detection sensitivity and dynamic range (Figure 2b,d). Single leukocyte‐associated secreted mass of each iSEAQ target was reported as the product of protein concentration and droplet volume (11.5 pL). The NE secretions were estimated with a linear regression estimate from a range of 1.4 fg/droplet to 38.3 fg/droplet, while GZB had a dynamic range of 0.4 fg/droplet to 11.5 fg/droplet. The FRET substrates designed for iSEAQ AMI profiling here were specific with minimal cross‐reaction between NE and the substrate targeting GZB (FRET substrate 2) and vice versa (Figure 2b,d).

Different concentrations of antibodies were encapsulated into droplets to create the standard curves shown in Figure 2e–g for CD66b, CD3, and CD31, respectively. The first‐order coefficients were extracted from the linear correlation trendlines (Table S4) to establish a conversion correlation between iSEAQ channel readings and the concentration of the panel targets. The selected panel for granulocytes (CD66b) and lymphocytes (CD3) showed strong correlations with clinical measurement profiles (Figure S8).

### 3.4 UMAP analysis of iSEAQ immune profiles across clinical cohorts

**FIGURE 3:**
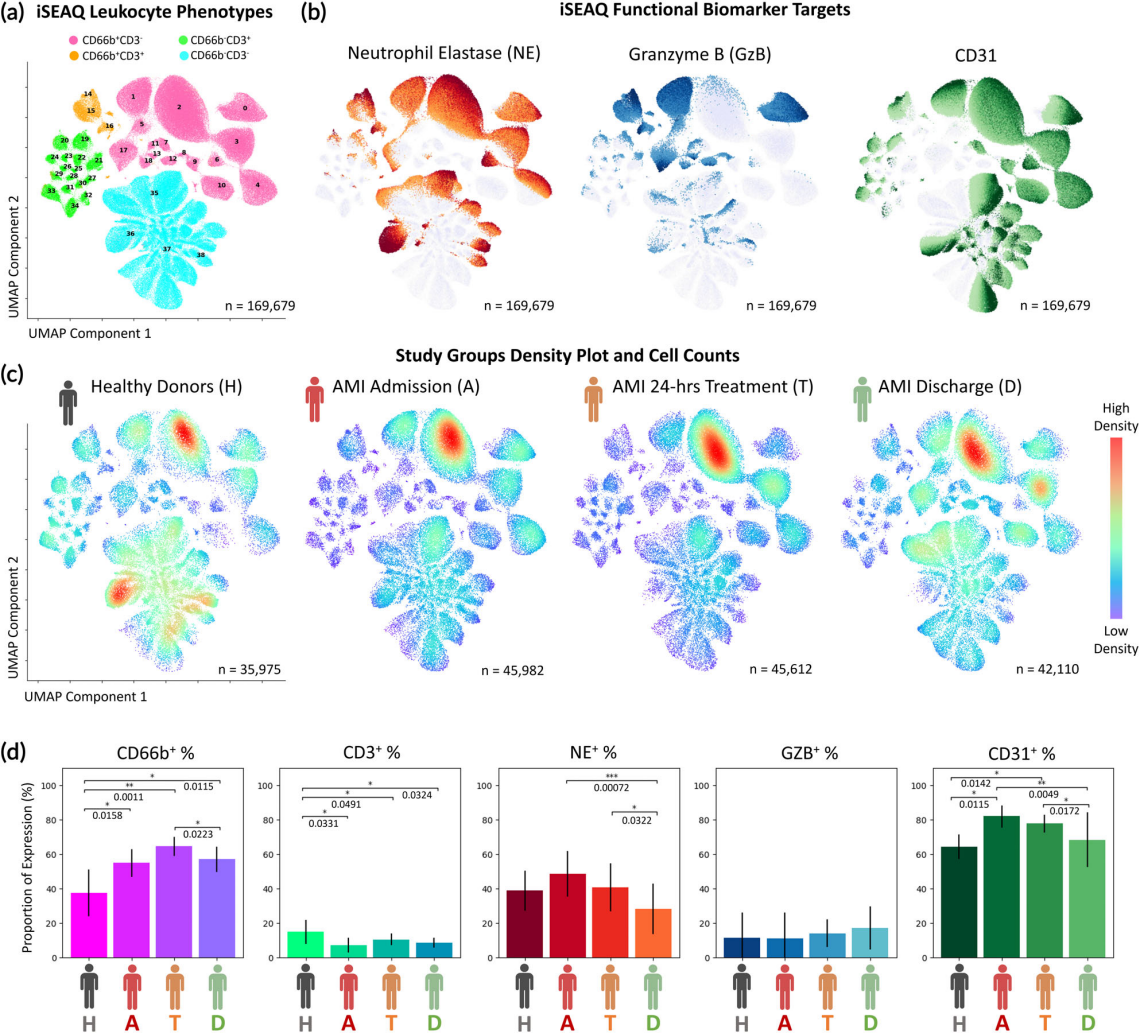
UMAP iSEAQ clusters, marker secretion and expression levels for four clinical cohorts. (a) UMAP clustering result of all single‐cell readings from the total 36 donors profiled in this study for a total of *n* = 169,679 single‐cell measurements, labeled by the phenotype classes (Pink: CD66b^+^CD3^−^, orange: CD66b^+^CD3^+^, green: CD3^+^, cyan: CD66b^−^CD3^−^). (b) shows the unsupervised clustering of NE, GZB and CD31 secretion and expression in the UMAP space highlighting distinct cell clusters in the UMAP result. (c) Density map distribution of cells for healthy (H), AMI admission (a), AMI 24‐h treatment (T) and AMI discharge (d), respectively. (d) shows the mean proportions of immune cells expressing surface antigen CD66b^+^, CD3^+^ and CD31^+^ and elevated enzyme NE and GZB secretions. All statistical analysis is based on *n* = 9 for each H, A, T and D clinical cohorts with two‐tailed paired t‐test performed for AMI patients and two‐tailed independent test performed between H and the rest. *, ** and *** denote significant *p*‐values of <0.05, 0.01 and 0.001, respectively.

We implemented a Uniform Manifold Approximation and Projection (UMAP) to visualize the immune profile of all single‐leukocyte droplets (*n* = 169,679) from all clinical cohorts and donors in this study (Figure 3) based on the 5‐channel iSEAQ values. Each point in the UMAP represents the projected data value of NE, GZB, CD31, CD66b and CD3 levels from one leukocyte. Figure 3a shows that the four iSEAQ immune phenotypes distribution in the UMAP space forms a total of 39 clusters from cluster 0 through 38. Figure 3b colors the entire UMAP by the expression level of NE, GZB and CD31 (high: dark, low: light). The expression heatmaps, together with the leukocyte phenotypes, indicate the immune cells and their corresponding functional secretion levels of NE and GZB, as well as surface expression levels of CD31. NE and GZB are generally highly secreted in different regions of UMAP while CD31 and NE show regions of overlapping expression in mostly CD66b^+^CD3^−^ clusters. Interestingly, from the parent UMAP projection, the four individual cohort density heatmaps show region of clusters and density suggesting these four groups (A, T, D, and H) had distinct immune signatures and profiles in iSEAQ assay and iSEAQ successfully captured the changing immune snapshots for AMI patients at different time points (Figure 3c).

The global expression and functional profiling of immune cells revealed that AMI patients had significantly elevated mean CD66b^+^ immune cell proportions, ranging from 56% to 63%, compared to 39% in healthy donors (Figure 3d). This is inversely true for CD3^+^ immune cell proportions, with AMI patients having fewer CD3^+^ immune cells. The number of immune cells secreting NE and expressing CD31 both showed a relatively elevated proportion in AMI onset during admission, while this proportion decreases after 24 h of treatment and decreases further upon discharge.

### 3.5 CD66b ^+^ cells: predominant role in immune response and enzyme secretion in AMI

**FIGURE 4:**
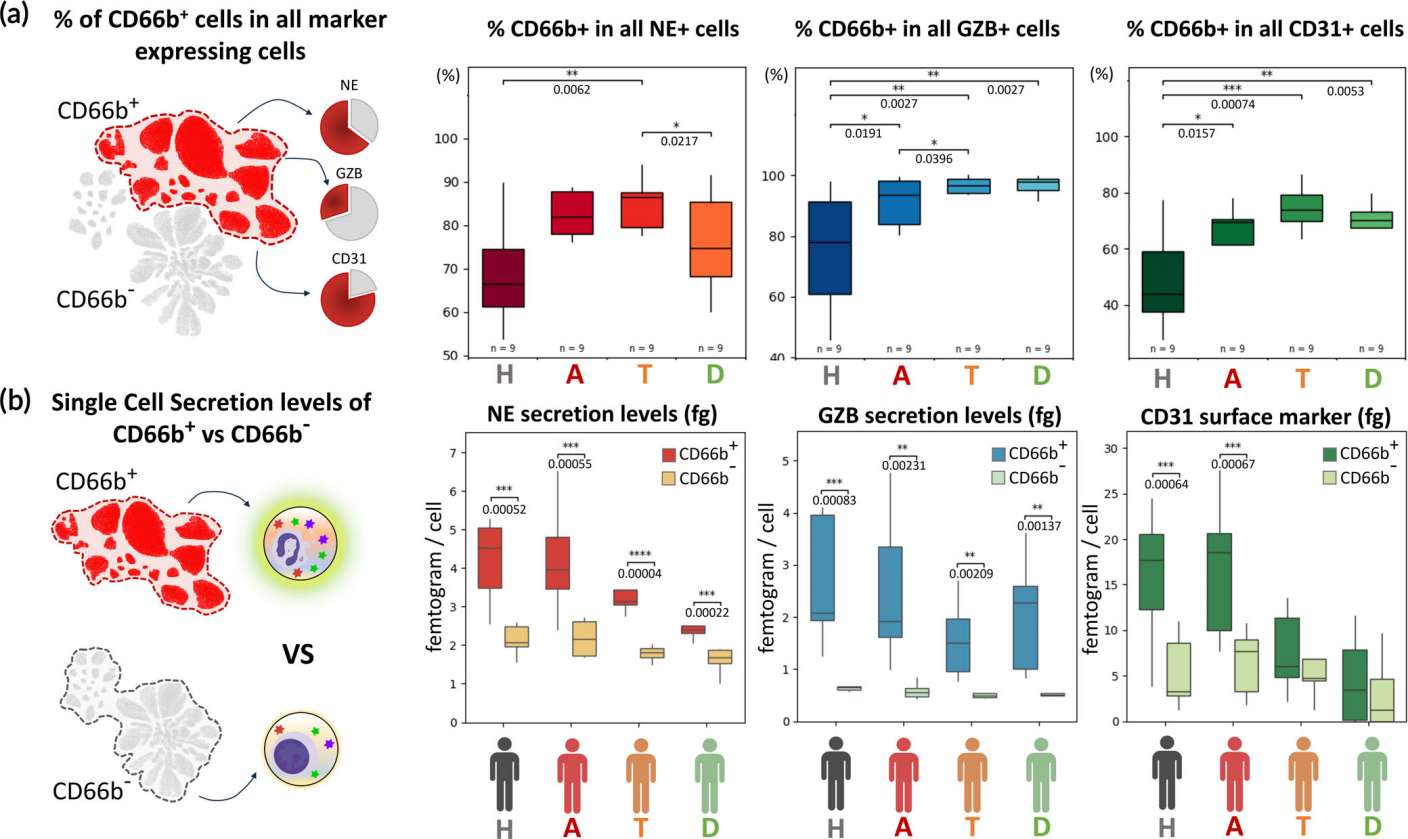
CD66b^+^ cells are a dominant phenotype for NE, GZB and CD31 immune activity in study cohorts. (a) Boxplots showing the percentage contributions of CD66b^+^ cells in all NE+, GZB+ and CD31^+^ immune cells, respectively. All statistical measurements in (a) with healthy donors were performed using a two‐tailed unpaired Student's t‐test, and AMI donors were analyzed using paired two‐tailed t‐test. (b) compares single‐cell mean NE and GZB secretion or CD31 surface marker expression levels of CD66b^+^ versus CD66b^−^ cells. All statistical analysis in (b) are performed using paired two‐tailed *t*‐test. *, **, *** represents *p* < 0.05, *p* < 0.01 and *p* < 0.001, respectively.

Further breakdown of the CD66b^+^ clustered data showed that it contributed disproportionately to all active immune cell activity of NE, GZB, and CD31 markers (Figure 4) in AMI. In contrast, CD66b^+^ cells in healthy controls (H) contributed significantly less enzyme secretion and immune activity for all profiled markers. In AMI, the contribution of CD66b^+^ cells to NE secretion was high on admission (A) and after 24 h (T), exceeding 80% in both, and decreased only at hospital discharge (D). The contribution of CD66b^+^ cells to GZB secretion is noteworthy, accounting for at least 90% of all GZB secretion in AMI patients. The contribution increased with time and accounted for 95% of all GZB secretion at the time of AMI discharge (D). This is a surprising finding suggesting that granulocytes are the dominant contributor to GZB secretions in AMI cohorts. It also suggests that CD66b^−^ cells, including CD3^+^ lymphocytes, do not actively secrete GZB in AMI patients. At the time of AMI discharge, the iSEAQ patient profiles indicate signs of incomplete inflammatory resolution, with elevated proportions of CD66b^+^ cells in active secretion. This acute inflammation is predominantly driven by CD66b^+^ cells, which are shown here for the first time.

Granularity of single‐cell secretion provides insights into quantifiable activity across different cell phenotypes. The iSEAQ assay also showed that the mean single‐cell secretion level of the CD66b^+^ population expressed was significantly distinct from CD66b^−^ cells (Figure 4b). The secretion levels tend to drop over the course of AMI treatment and at discharge time points. There is an improvement in acute inflammation for the duration of treatment and discharge of AMI patients, which is likely modulated by clinical interventions. Critically, these changes in immune cell secretion levels were observed over 2 to 4 days from admission to discharge, a period during which iSEAQ immune profiling can clearly distinguish them.

### 3.6 Granular analysis of iSEAQ profiling in major immune cell clusters

**FIGURE 5:**
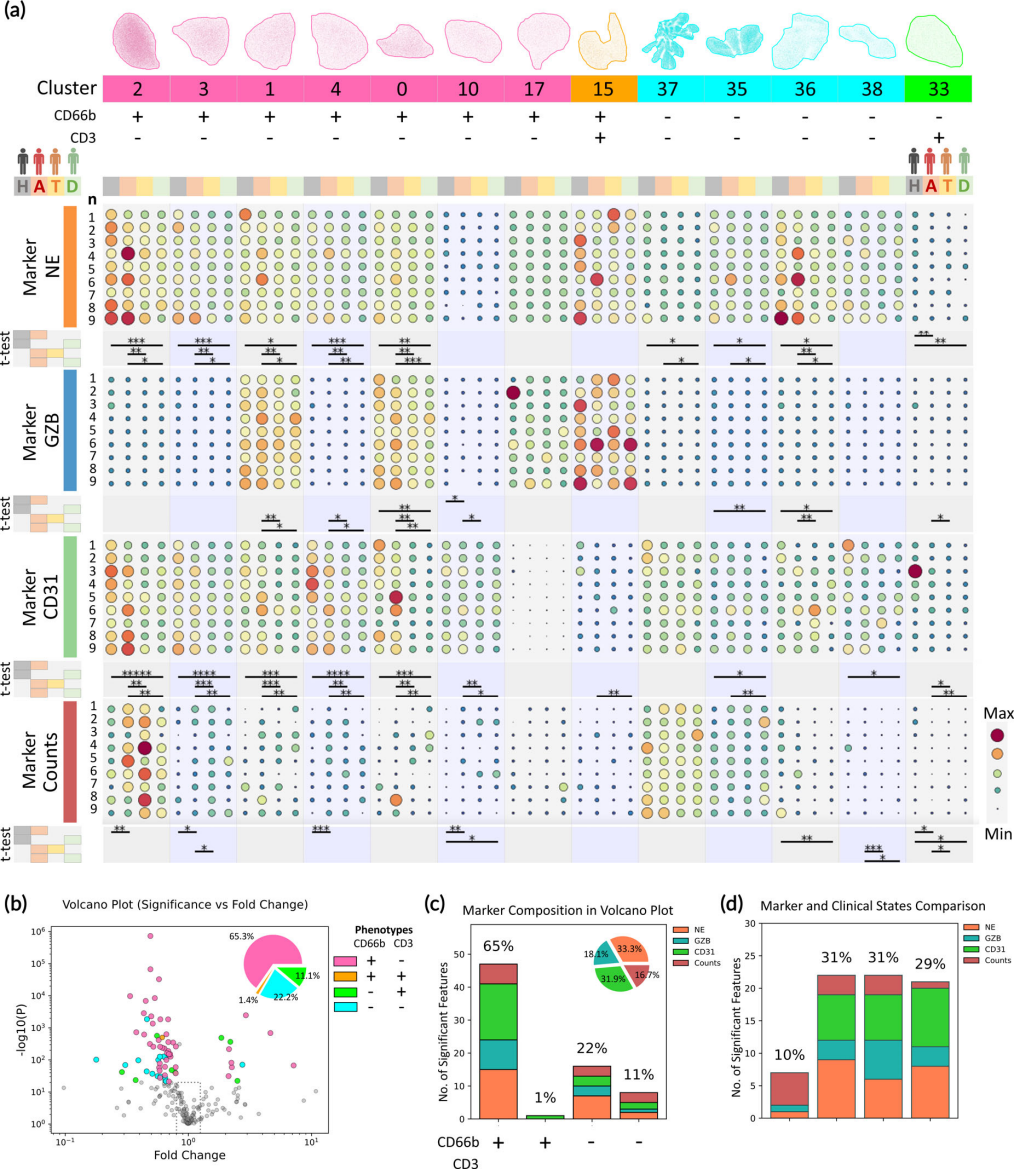
iSEAQ UMAP cluster breakdown for individual donors by CD66b and CD3 phenotypes. The dotplot in (a) shows secretion levels of NE, GZN and CD31 of the predominant immune phenotypes grouped by the selected 13 clusters profiled by iSEAQ. The data is normalized and scaled by dot size and color. The 4th marker “counts” shows the number of immune cells within the clusters. Each cluster consists of contributions from 36 samples grouped by each cohort of healthy (H), AMI admission (a), AMI 24‐h treatment and AMI discharge with *n* = 9 for each group. These groups are represented by the color gray, beige, yellow and green, respectively. Statistical analysis of the groups was performed using t‐test for H vs. A, H vs. D, A vs. T and A vs. D. All statistical measurements with healthy donor were performed using an unpaired Student's t‐test (black lines) and AMI donors were analyzed using a paired Student's t‐test (green lines). *, **, ***, ****, ***** represents *p* < 0.05, *p* < 0.01, *p* < 0.001, *p* < 0.0001, *p* < 0.00001, respectively. (b), Volcano plots summarizing the statistical robustness of four iSEAQ key single‐cell features, namely cell counts, NE level, GZB level, and CD31 level. The features above the black dotted line are significantly different (*p* < 0.05) and the features outside the vertical dotted lines are those that showed increased fold changes (p < 0.05). Features that were of interest were color‐coded to identify the contributions by phenotype, namely CD66b^+^CD3^−^ (pink), CD66b^+^CD3^+^ (orange), CD66b^−^CD3^+^ (green) and CD66b^−^CD3^−^ (cyan). The percentage contribution to significance by phenotype is shown in the pie graph. (c) counts and groups the number of significant features by phenotype and is broken down into the proportions of markers. (d) shows the distribution of significant features and the iSEAQ marker breakdown by t‐test groups.

In our analysis, detailed in Figure 5a, we focused on the 13 most significant clusters identified through UMAP, each representing at least 1% of the total immune cell count (*n* = 167,679). These clusters were ranked and accounted for a substantial 89% of all immune cells profiled by iSEAQ (see Figure S9). Notably, the CD66b^+^CD3^−^ cluster, making up 47.7% of these cells, was the most prominent, with cluster 2 alone comprising 20.8% of all cells profiled. In contrast, CD66b^−^CD3^−^ leukocytes constituted 38.1% of the profiled cells.

A deeper analysis revealed that CD66b^+^ cells significantly differentiated the study groups, as evidenced in the volcano plots (Figure 5b,c). These cells were involved in nearly twice as many significant comparisons (65%) as CD66b^−^ cells (35%). In terms of cellular activity, CD66b^+^ cells exhibited a two‐fold increase in significant activity relative to their cell counts when compared to CD66b^−^ cells. This finding positions CD66b as a particularly significant marker for peripheral blood profiling in AMI, underscoring its potential as a key indicator in clinical immune response profiling.

Cluster 15 shows a CD66b^+^CD3^+^ cell population with the highest granzyme B secretion among all clusters. This population expresses both granulocyte (CD66b) and lymphocyte (CD3) markers and is likely a granulocyte phenotype with the presence of a T cell receptor (TCR)‐based expression. Kerstin et al. first showed the presence of a 5–8% granulocyte sub‐population with these receptors, and the percentage of CD66b^+^CD3^+^ phenotype in all CD66b^+^ cells in this study is 6.6%, which agrees with the literature.^38^ This population also constitutes 17.5% of all GZB secretions and activity across all sample cohorts, a rather significant proportion of GZB activity.

In comparing the various sample groups in Figure 5d, the H vs. A group showed the largest significance in cell counts, suggesting that AMI patients on the onset of admission could have an initial increase in immune cells. This is likely a normal immune response. In comparison, the H vs. D, A vs. T, and A vs. D t‐test groups showed the largest differences, with 31%, 31%, and 29% significant features, respectively.

### 3.7 iSEAQ immune profiling and linear regression model for AMI prognostication

There is increasing evidence supporting the connection between immune response and cardiac diseases, with the use of blood count parameters as a more effective prognostic indicator compared to conventional cardiac markers such as creatine kinase MB (CK‐MB) and troponin I.^39^ However, white blood cell count is not a time‐resolved indicator for immune response given that immune activity can change within minutes of exposure to external stimuli. Thus, iSEAQ immune response profiling can potentially augment the current prognostication models in AMI with direct measurement of single‐cell activity and function.

**FIGURE 6:**
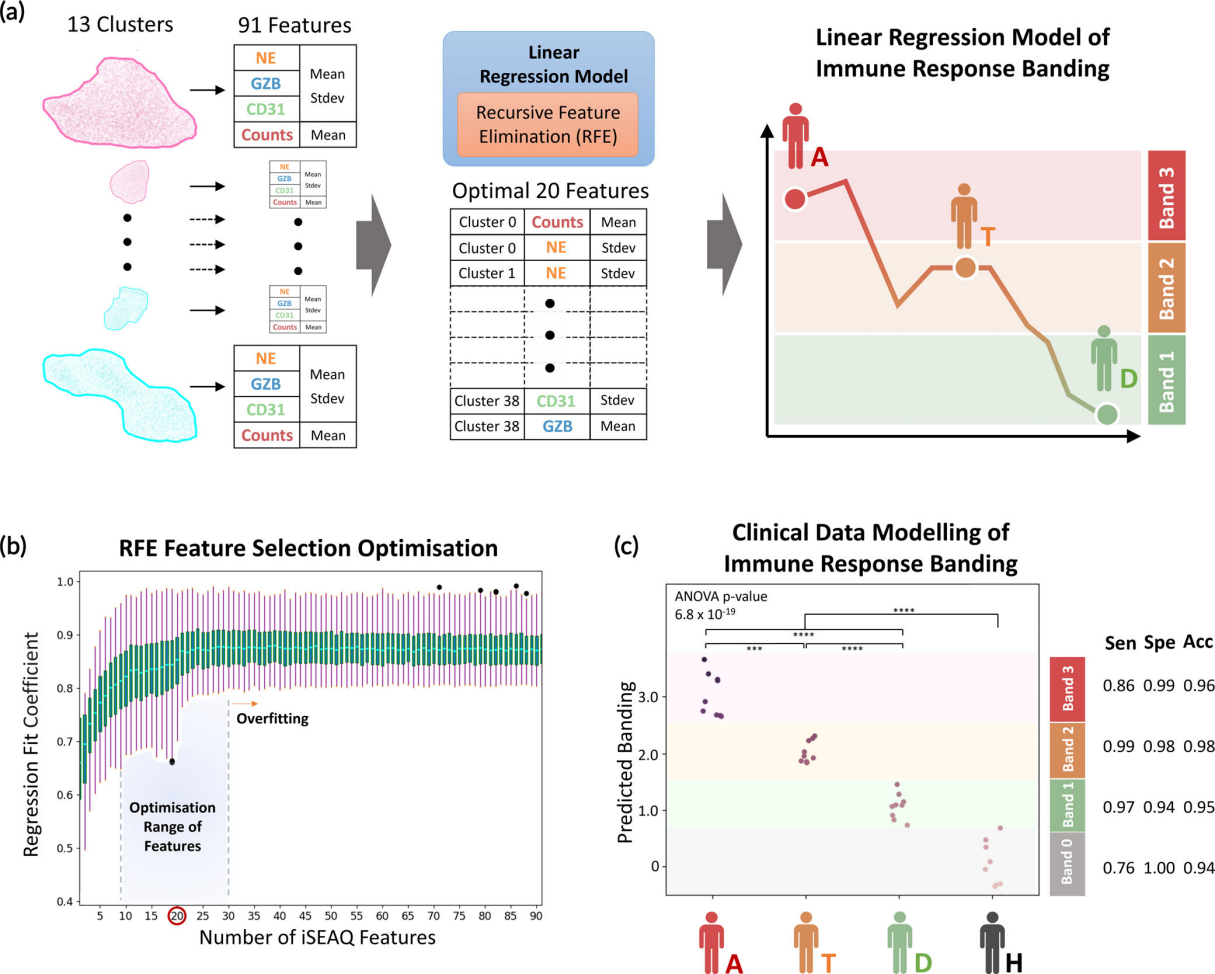
Developing a prognostic and correlative model using iSEAQ features for AMI. (a) shows the use of 91 immune features from the 13 significant clusters in UMAP space to develop a linear regression model for the proposed banding of AMI patients in the respective clinical progression from admission to discharge. A recursive feature elimination (RFE) method is used to reduce the features to an optimal 20 features for training of the model. (b) The optimization data of RFE using the linear best fit coefficient for the training data is presented in a boxplot. The plateau of regression coefficient starts around 30 features, denoting overfitting of data. In (c), using 20 selected features, a predictive linear regression model was developed and tested for all samples, showing high sensitivity (Sen), specificity (Spe), and accuracy (Acc) for the banding of AMI patients. One‐way ANOVA was performed on the data with individual two‐tailed Student's t‐tests performed (Table S6). *** and **** represent *p*‐value <0.001 and <0.0001, respectively.

As a preliminary proof‐of‐concept, as shown in Figure 6a, the features in iSEAQ are used to develop a linear regression model to test the concept of an immune response banding in AMI patients. A recursive feature elimination method was used to select the optimal features for the model. As seen in Figure 6b, as the number of features increases, the model fitting coefficient increases and plateaus at around 30 features, suggesting the possibility of overfitting with additional features. An optimal number of 20 iSEAQ features was used to develop the linear regression model for AMI patient banding (Table S5).

Each data point in Figure 6c is a predicted clinical value. A “blind” sample was selected, and bootstrapping methods were performed on the remaining sample pool to calculate the coefficients and the model that is used to predict the “blind” sample. The model gives high predictive specificity, sensitivity, and accuracy for the respective bands. The suggested banding corresponds to a specific progression of AMI patients in this cohort study. The algorithm modeling and prediction is presented in Section 2 and Figure S10.

The immune response banding shows the potential for the use of a model algorithm to be developed further in expanded clinical studies. A one‐way ANOVA test was performed and showed distinct significance between all groups (*p* = 6.8 *10^−19^). Interclinical group statistics were performed and the model performed clear clustering with distinction among each other using both the t‐test and the Tukey HSD test as shown in Table S6.

## 4 DISCUSSION

Our innovative iSEAQ assay represents a significant advancement in characterizing immune responses in AMI. Quantitatively, iSEAQ can detect femtograms of secreted enzymes and surface antigens from thousands of live leukocytes within just 30 min from a mere 20 μL of whole blood. Qualitatively, iSEAQ provides unprecedented granularity in tracking the rapidly evolving immune landscape in AMI patients by demonstrating: (1) the inflammatory burst in AMI triggered by ischemia and reperfusion through increased inflammatory markers, (2) the trajectory of inflammation through distinct immune profiles in discharged AMI patients compared to admission and healthy controls, and (3) a predominance of CD66b^+^ cell‐driven inflammatory response (through disproportionate CD66b^+^ cell counts) during AMI, persisting even at hospital discharge.

The rationale for the benefit of iSEAQ in AMI lies in the notion of inflammation and immune activation driving clinical outcomes leading to and following AMI. This is particularly important in the acute post‐AMI phase, where targeting the early inflammatory response has not seen any significant clinical breakthrough, partly due to difficulties in identifying the right targets at the right time.^40^ Polymorphonuclear cells contribute to the “ugly” side of inflammation in AMI, given the sudden burst of immune activity in early stages of AMI.^40^ The iSEAQ's ability for real‐time, granular profiling of immune activation may advance the field of AMI therapeutics by identifying novel targets. Indeed, by demonstrating the predominance of CD66b^+^ cell‐driven early inflammatory response post‐AMI, it highlighted the critical role of these cells in the pathophysiology and positioned them as potential therapeutic targets.

Our finding of peripheral CD66b^+^ cells being the primary source of Granzyme B (GZB) secretion in AMI, which runs contrary to the established understanding of GZB as predominantly lymphocyte‐derived,^41^ is worth highlighting. Zeglinski et al.^42^ in a recent review suggest that granzymes should no longer be considered exclusively as cytotoxic T‐cell or NK cell specific markers. Pre‐clinical models investigating roles for GZB in cardiovascular diseases are limited; however, those published have indicated novel roles for plasma GZB correlating with vascular tissue remodeling.^43^ Functional roles of GZB in CD66b^+^ granulocyte population remains poorly understood,^44^, ^45^ and have not been reported for AMI. Amandine et al. discovered an increase in GZB secretion for tumor‐derived neutrophils^46^; Prior studies have also shown granulocytes secretion of GZB during infection.^46^, ^47^, ^48^ Shen et al. performed tissue histology and showed that CD68 immune cells secrete GZB which cleaves VE‐cadherin to disrupt endothelial junctions and is known to remodel ECM and activate pro‐inflammatory cytokines, thereby promoting vascular leak and cardiac fibrosis.^49^ This study links GZB to non‐cytotoxic lymphocyte secretion of GZB. Yet, our study here investigates GZB secreted by granulocytes of AMI patients from onset to recovery, albeit with increasing proportions of GZB secretions up to >95%.

Emerging evidence shows that CD66b^+^ granulocytes (neutrophils) can be “reprogrammed” by inflammatory cues to both express and secrete GZB in a perforin‐independent manner. In tuberculosis granulomas, naïve blood neutrophils lack GZB, but exposure to Mycobacterium tuberculosis components or LPS induces GZB transcription and release, as detected by intracellular staining and ELISPOT assays.^48^ Similarly, in tumor models, tumor‐associated neutrophils upregulate GZB when stimulated with a cytokine mix (IL‐2, IL‐12, IL‐21) alone or combined with a TLR4 agonist, demonstrating that innate (TLR) and adaptive (cytokine) signals converge to trigger neutrophil GZB production.^46^ Once secreted, neutrophil‐derived granzyme B could potentially function to degrade extracellular proteins (e.g., fibronectin, vitronectin) and process pro‐IL‐1α/IL‐18, thereby amplifying local inflammation and tissue remodeling.^45^ This differs from granzyme‐perforin secretions by T cells, which act on intracellular processes to kill targets. These findings highlight a novel mechanism whereby pro‐inflammatory microenvironments induce CD66b^+^ cells to contribute to GZB‐mediated pathology, suggesting the need for further research into granulocyte functionality in AMI, facilitated by iSEAQ's single‐cell functional profiling capabilities.^50^, ^51^, ^52^

To contextualize the current data in iSEAQ, it is important to understand the role of neutrophils in AMI and their contributions compared with those of monocytes, macrophages, and lymphocytes. Neutrophils infiltrate the infarct within hours, releasing ROS, proteases (e.g., elastase, MMP‐9) and neutrophil extracellular traps that drive initial tissue injury and amplify inflammation but later support the resolution through apoptotic clearance signals often occurring around 24 h by macrophages.^11^ By days 1–2, monocytes arrive at the site and differentiate into pro‐inflammatory M1 macrophages, secreting IL‐1β, TNF‐α and IL‐6 to clear debris, then transition to M2 macrophages that produce the anti‐inflammatory cytokine IL‐10, TGF‐β and VEGF to promote scar formation and angiogenesis^53^; lymphocyte CD4^+^ T cells accumulate by days 5–7 where T helper cells secrete IFN‐γ and IL‐17 to sustain inflammation, whereas Th2/Treg cells release IL‐10 and TGF‐β to restrain damage and foster repair.^54^ In this context, neutrophils are clearly the front‐line immune cells for acute conditions and GZB could potentially support the initial response. An interesting link to granzyme B is shown that basophils release GZB upon acute inflammation such as asthma^55^ and thus, it is plausible that neutrophil secretion of GZB function may not be specific to AMI but a huge contributor as an acute response secretion molecule.

iSEAQ's advantages over standard proteomic methods are clear: it measures live leukocyte enzyme activity directly but does so more rapidly and sensitively than existing assays. iSEAQ profiles immune cells directly from blood to results with at least two orders of magnitude faster than existing single‐cell secretion assays such as Isoplexis^56^ and Elispot^57^ assays, which require at least 24 h of sample prep and analysis. Moreover, its rapid sample processing closely approximates in vivo conditions, an improvement over FACS, intracellular staining (ICS) or micro‐well based methods, which require sample manipulation exposing leukocytes to non‐physiological stresses prior to immune profiling.^58^, ^59^ Numerous studies have shown that DLD sorting exerts minimal forces (Reynold's number <1) on cells, resulting in >95% viability.^60^, ^61^, ^62^, ^63^ Furthermore, Campos‐González et al. showed T cells processed through DLD at much higher pressures (690 mBar) exhibit lower activation marker expression than those subjected to standard RBC‐lysis/Ficoll protocols,^30^ suggesting minimal inadvertent activation by our sorting method. iSEAQ operates at ~3 μL/min (450 mBar), producing cell velocities approximating physiological shear levels, and comparable immune‐cell DLD sorters have demonstrated >95% viability after 30 min with no loss of downstream function.^64^ While in this study, 30 min incubation time is used, it is possible to reduce the incubation time down to 15 min for a detectable FRET signal.

Through granular characterization of inflammation and immune activation, this brings us closer to the ideal state of personalized medicine where we can select AMI patients with large inflammatory burden, focus on a specific causal target, time our treatment according to the immune activation and follow through with a tapering regimen based on response. We acknowledge several limitations: our panel was confined to five fluorescence channels and two phenotypic probes, and we did not include pharmacological inhibition studies or GZB gene expression data, which would have provided deeper mechanistic insights. We developed iSEAQ as an immune‐secretion diagnostic platform, and future study adding single‐cell transcriptomics could yield further discoveries. Beyond AMI, iSEAQ has potential applications in sepsis, autoimmune diseases, and cancer.^46^, ^65^, ^66^ Overall, our study advances immune profiling by delivering a rapid tool for assessing immune function and phenotype, crucial for timely clinical decision‐making and effective therapeutic interventions.

## 5 CONCLUSION

In conclusion, the iSEAQ assay is a powerful tool for studying immune responses in AMI patients. This innovative assay enables quantification of femtograms of secreted enzymes and surface antigens from live leukocytes in just 30 min, providing a detailed immune profile at single‐cell resolution. The study reveals a dynamic and persistent inflammatory response in AMI patients, driven significantly by CD66b^+^ cells, even at the time of discharge. Notably, peripheral CD66b cells are identified as the dominant source of Granzyme B (GZB) secretion, challenging conventional knowledge. This novel observation emphasizes the critical role of CD66b^+^ cells in AMI and underscores the need for a comprehensive tool to elucidate their real‐time single‐leukocyte functional enzyme secretion and surface antigen expression. The findings invite further exploration of immune responses in longitudinal studies and highlight iSEAQ's versatility in investigating diseases involving immune activation and inflammation.

## AUTHOR CONTRIBUTIONS

Shir Lynn Lim, Kerwin Kwek Zeming, Jongyoon Han designed the clinical experiments. Shir Lynn Lim applied for IRB and oversaw the clinical study. Shir Lynn Lim., Nicholas W. S. Chew and Kai Lee Woo recruited the patients and collected the samples. Kerwin Kwek Zeming, Ri Lu and Jongyoon Han conceived, designed and fabricated the DLD and iSEAQ integrated device. Ri Lu and Kerwin Kwek Zeming optimized and performed the iSEAQ experiments. Ri Lu, Elizabeth Lee, Kerwin Kwek Zeming designed, assembled, and calibrated the optical and data acquisition setup. Ri Lu designed and calibrated the iSEAQ panels. Ka‐Wai Cheung, Ri Lu, Kerwin Kwek Zeming perform the iSEAQ raw data analysis and compensation. Ri Lu coded the algorithms for signal processing and visualization. Ri Lu, Kerwin Kwek Zeming, Lih Feng Cheow, Shir Lynn Lim discussed the clinical data presentation. Ri Lu, Kerwin Kwek Zeming, Shir Lynn Lim and Jongyoon Han wrote the manuscript. All authors reviewed and approved of the manuscript prior to submission.

## FUNDING INFORMATION

This research is supported by the National Research Foundation, Prime Minister's Office, Singapore under its Campus for Research Excellence and Technological Enterprise (CREATE) programme, through funding of the Intra‐Create Grant NRF2020‐ITS006‐0013 and Singapore‐MIT Alliance for Research and Technology (SMART): Critical Analytics for Manufacturing Personalized‐Medicine (CAMP) Inter‐Disciplinary Research Group.

## CONFLICT OF INTEREST STATEMENT

Authors declare no competing interests.

## ETHICS STATEMENT

The study was conducted in accordance with the Declaration of Helsinki, and the protocol for the collection of all samples was approved by the local Institutional Review Board (DSRB 2021/00246).

## CONSENT FOR PUBLICATION

All participants (patients and healthy controls) provided written informed consent to the study.

## Supporting information


**DATA S1.** Supporting Information.

## Data Availability

The datasets generated during and/or analyzed during the current study are available from the corresponding author upon reasonable request.
