# Supplementary material for: Ex vivo single‐cell profiling of acute myocardial infarction patients reveals disproportionate CD66b + cell secretion response
Source: Bioeng Transl Med. 2025 Jul 7;10(6):e70043. doi: 10.1002/btm2.70043 (PMC12617547; doi:10.1002/btm2.70043)
Supplement: Supplementary file 1 — DATA S1. Supporting Information. [file BTM2-10-e70043-s001.docx]

**Supplementary Data**

**Ex Vivo Single-cell Profiling of Acute Myocardial Infarction Patients Reveals Disproportionate CD66b+ Cell Secretion Response**

Kerwin Kwek Zeming^1,#^, Ri Lu^1,2,#^, Elizabeth Lee^1^, Ka-Wai Cheung^1^, Nicholas WS Chew^3^, Kai Lee Woo^3^, Lih Feng Cheow^1,4^, Jongyoon Han^1,5,6,*^, Shir Lynn Lim^7,8,9,*^

^1^ Singapore-MIT Alliance for Research and Technology, Critical Analytics for Manufacturing of Personalised Medicine

^2^ National University of Singapore, Graduate School for Integrative Sciences and Engineering

^3^ National University Health System, National University Hospital

^4^ National University of Singapore, Department of Biomedical Engineering

^5^ Massachusetts Institute of Technology, Department of Electrical Engineering and Computer Science

^6^ Massachusetts Institute of Technology, Department of Biological Engineering

^7^ Department of Cardiology, National University Heart Centre Singapore

^8^ Yong Loo Lin School of Medicine, National University of Singapore

^9^ Pre-hospital Emergency Research Centre, Duke-NUS Medical School

#Co-First Authors

* Co-corresponding Authors:

Asst. Prof Lim Shir Lynn (M.D.)

shir.lynn.lim@nus.edu.sg

Prof Jongyoon Han
[jyhan@mit.edu](mailto:jyhan@mit.edu)

**Supplementary Table S1.** Technical comparison of iSEAQ with previous work

|  | Previous Work[1]  (Main manuscript ref. 29) | iSEAQ (This study) |
| --- | --- | --- |
| Microscope | Leica DMi8 fluorescence microscope. Direct microscope coupling using c-mount to photomultiplier tube (PMT). **No light focusing to PMT.** | Custom optical stage setup for optimised field of view and higher optical coupling efficiency. **All light to avalanche photodiodes (APD) were focused using lenses.** |
| Detector | PMT  Hamamatsu H9306-03 | APD  Hamamatsu C12703-01 |
| Detector frequency bandwidth | DC to 20 kHz | **DC to 100 kHz** |
| Data Sampling | 12.5kHz | **200kHz** |
| Realtime Data Processing | No | **Yes** |
| Software | Matlab for post-processing | Python for Graphical User Interface, data parsing, data processing and data visualisation. |
| Light Source | White LED | Blue Laser (488nm) 100mW |
| Fluorescence Channels | 4 channels  (3 Biomarkers, 1 Nucleus Stain) | 5 channels  (5 biomarkers) |
| Biomarkers | Neutrophil Elastase,  Granzyme B,  Metalloproteinase (MMP) | Neutrophil Elastase,  Granzyme B,  **CD31,**  **CD66b,**  **CD3** |
| Profiled immune cells | No-discrimination | **Granulocytes, lymphocytes and negative subtypes** |
| Cell in Droplet Detection | Hoechst stain (blue channel) | Label-free FTL detection |
| Droplet Detection | No | **Yes, real-time** |
| Droplet incubation  (incubation time range) | 60min  (10 – 120mins) | 30 mins  (5 – 30mins) |
| Microfluidics | Same | Same |
| Processing Pressure | 300mBar | 450mBar |
| Clinical Cohort | Acute Heart Failure  (n = 5) | Acute Myocardial Infraction  (n = 9) |
| Observation Window | Admission, discharge | Admission, treatment and discharge |
|  |  |  |

**Supplementary Table S2.** AMI patient (n = 9) clinical data

| Subject ID | Age | Gender | Type of STEMI | Duration of stay (days) | Remarks |
| --- | --- | --- | --- | --- | --- |
| AMI-001 | 54 | Male | Anterior | 2 |  |
| AMI-002 | 45 | Male | Anterior | 3 |  |
| AMI-003 | 44 | Male | Anterior | 2 |  |
| AMI-004 | 59 | Male | Anterior | 2 |  |
| AMI-005 | 44 | Male | Inferior | 4 |  |
| AMI-006 | 45 | Male | Inferior | 4 | Anterior STEMI, VF collapse Day 2 |
| AMI-007 | 55 | Male | Inferior | 2 |  |
| AMI-008 | 65 | Male | Inferior | 2 |  |
| AMI-009 | 52 | Male | Inferior | 2 |  |

**Supplementary Table S3.** iSEAQ phenotypes and their corresponding leukocyte linages

| iSEAQ phenotype | CD66b^+^CD3^-^ | CD66b^+^CD3^+^ | CD66b^-^CD3^+^ | CD66b^-^CD3^-^ |
| --- | --- | --- | --- | --- |
| Key leukocyte linage  (Referenced from  existing studies) | Granulocyte[2]:   - Neutrophil - Basophil - Eosinophil | Granulocyte:   - Neutrophil[3] | T lymphocyte[4] | B lymphocyte  NK  Monocyte |
| Remarks | Predominant in CD66b^+^ | Rare in CD66b^+^ |  |  |

**Supplementary Table S4.** Coefficients to compute single droplet target concentration from raw readings of 5 iSEAQ channels

|  | **NE** | **GzB** | **CD66b** | **CD3** | **CD31** |
| --- | --- | --- | --- | --- | --- |
| **k** | 1382.1 | 2363.0 | 0.91 | 0.73 | 0.77 |
| **b** | -2003 | -814 | 1.87 | 2.51 | 2.62 |

NE, GZB ----------------- $y=kx+b$

CD66b, CD3, CD31 ---- ${log}_{10}\left( y \right)=k*{log}_{10}\left( x \right)+b$

**Supplementary Table S5.** 20 features selected from linear regression RFE model

|  | **Cluster** | **Marker** | **Statistics** | **CD66b** | **CD3** | **coefficient weight** |
| --- | --- | --- | --- | --- | --- | --- |
| 1 | 0 | NE | Stdev | + | - | 0.411 |
| 2 | 1 | NE | Stdev | + | - | 0.694 |
| 3 | 1 | Counts | Mean | + | - | 0.605 |
| 4 | 1 | CD31 | Stdev | + | - | 0.625 |
| 5 | 2 | CD31 | Mean | + | - | -0.723 |
| 6 | 3 | GZB | Stdev | + | - | -0.427 |
| 7 | 4 | Counts | Mean | + | - | 0.400 |
| 8 | 4 | CD31 | Mean | + | - | -0.320 |
| 9 | 10 | GZB | Stdev | + | - | 0.751 |
| 10 | 10 | Counts | Mean | + | - | -0.332 |
| 11 | 10 | NE | Mean | + | - | 0.203 |
| 12 | 15 | NE | Stdev | + | + | 0.484 |
| 13 | 15 | GZB | Mean | + | + | -0.249 |
| 14 | 33 | CD31 | Stdev | - | + | 0.330 |
| 15 | 35 | GZB | Stdev | - | - | -0.690 |
| 16 | 37 | NE | Stdev | - | - | -0.417 |
| 17 | 37 | Counts | Mean | - | - | 0.253 |
| 18 | 37 | CD31 | Stdev | - | - | -0.203 |
| 19 | 38 | CD31 | Stdev | - | - | 0.298 |
| 20 | 38 | GZB | Mean | - | - | 0.461 |

The linear regression model in Equation 1 is based on:

$Y_{i}=\beta_{0}+\beta_{1}X_{1}+\beta_{2}X_{2}+\ldots+\beta_{n}X_{n}$ ------ Equation 1.

Where $Y$ denotes the predicted $i$ banding for $i=0, 1, 2 or 3$ and X denotes the iSEAQ features with coefficients $\beta_{n}$ where $n$ denotes the coefficient number and more importantly describing the contribution to the band.

**Supplementary Table S6.** Tukey HSD test with confidence level of 95% for the various combinations of groups with corresponding two-tailed paired and independent t-test and significance. With *** and **** representing p < 0.001 and p < 0.0001 respectively.

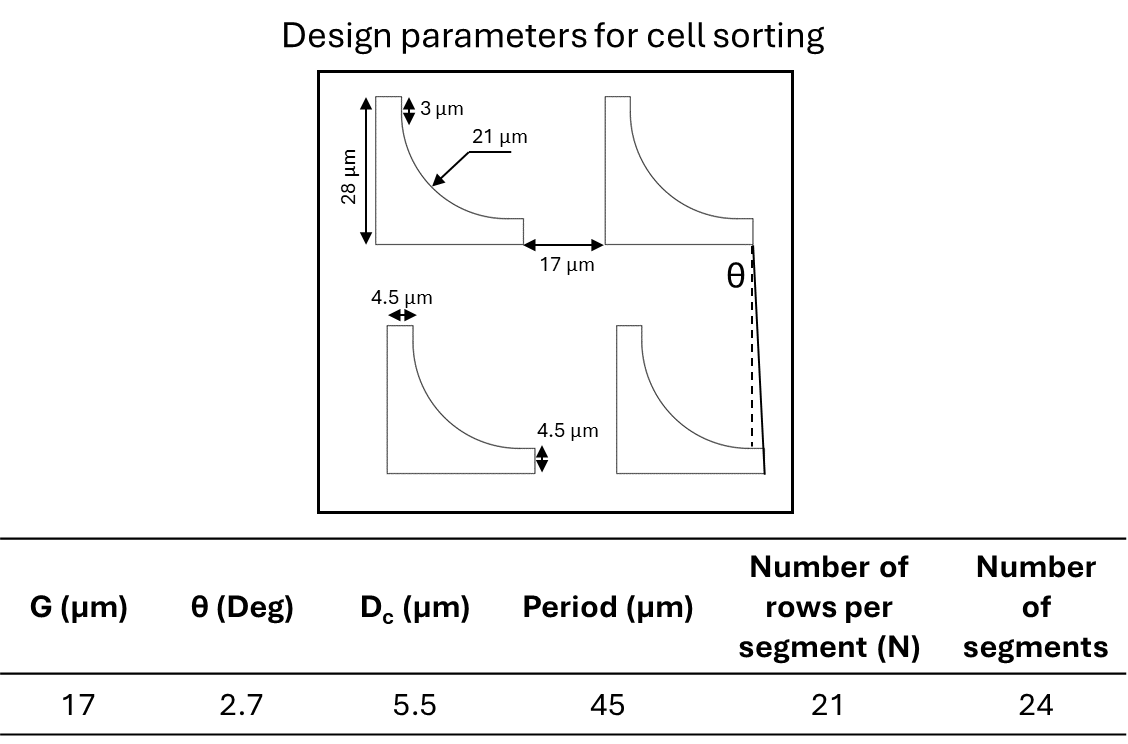


**Supplementary Figure S1.** Design parameters for L-shaped pillars in DLD immune cell sorting[1]. The tables show the DLD parameters with D_c_ specifying the critical cut-off size for cell sorting based on the empirical formula by Davis et al.[5], D_c_ = 1.4·G·(tanθ)^0.48^, where D_c_ defines the cut-off cell size, G defines the gap spacing between pillars and θ describes the slope or gradient of the pillar array. The specifications and drawings here are adapted from Zeming et al. Supplementary Figure S8[1] under CC-BY-NC-ND 4.0.


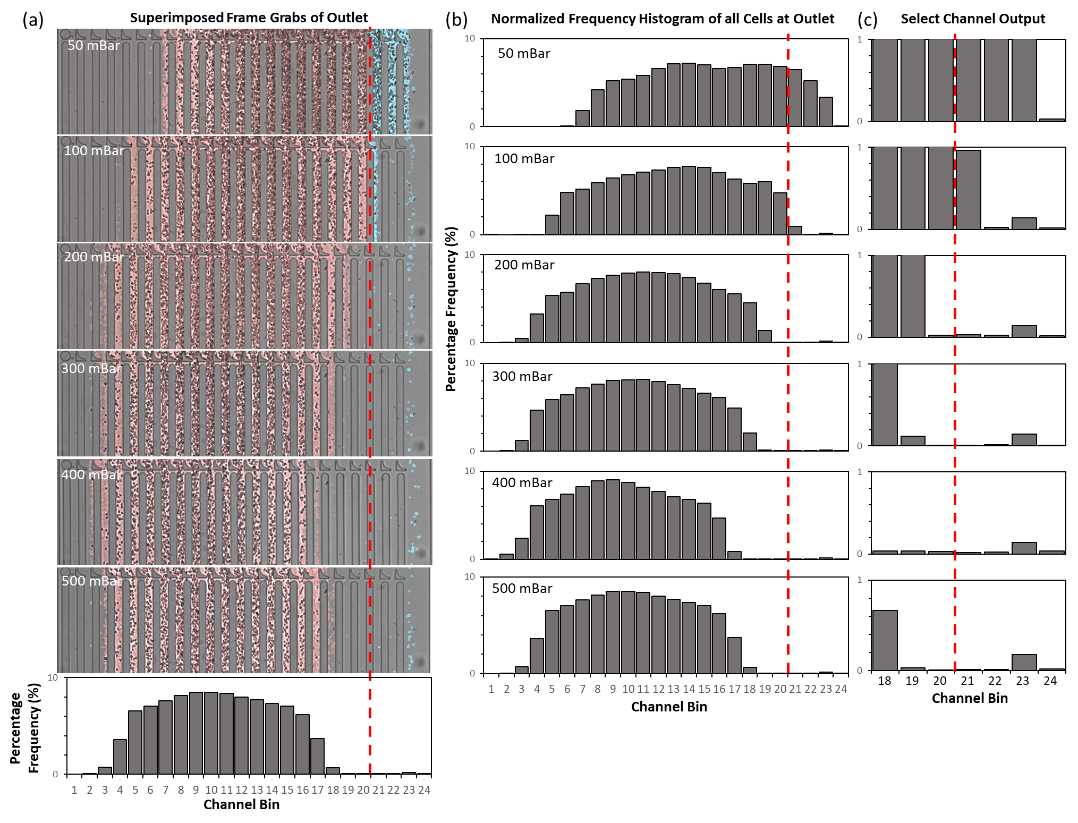


**Supplementary Figure S2.** Microfluidic DLD-L device output showing cell sorting of blood cells which are characterised at varying pressures from 50 – 500 mBar (a). (b) tabulates the normalized frequency distribution percentage plots of all cells passing through the channel bins with (c) showing the magnified view of the channel bins 18 – 24. The red dotted line denotes the bifurcation position of the sorted channels. Reproduced from Reference1 by Zeming et al.[1] under CCBY-NC-ND 4.0 license.


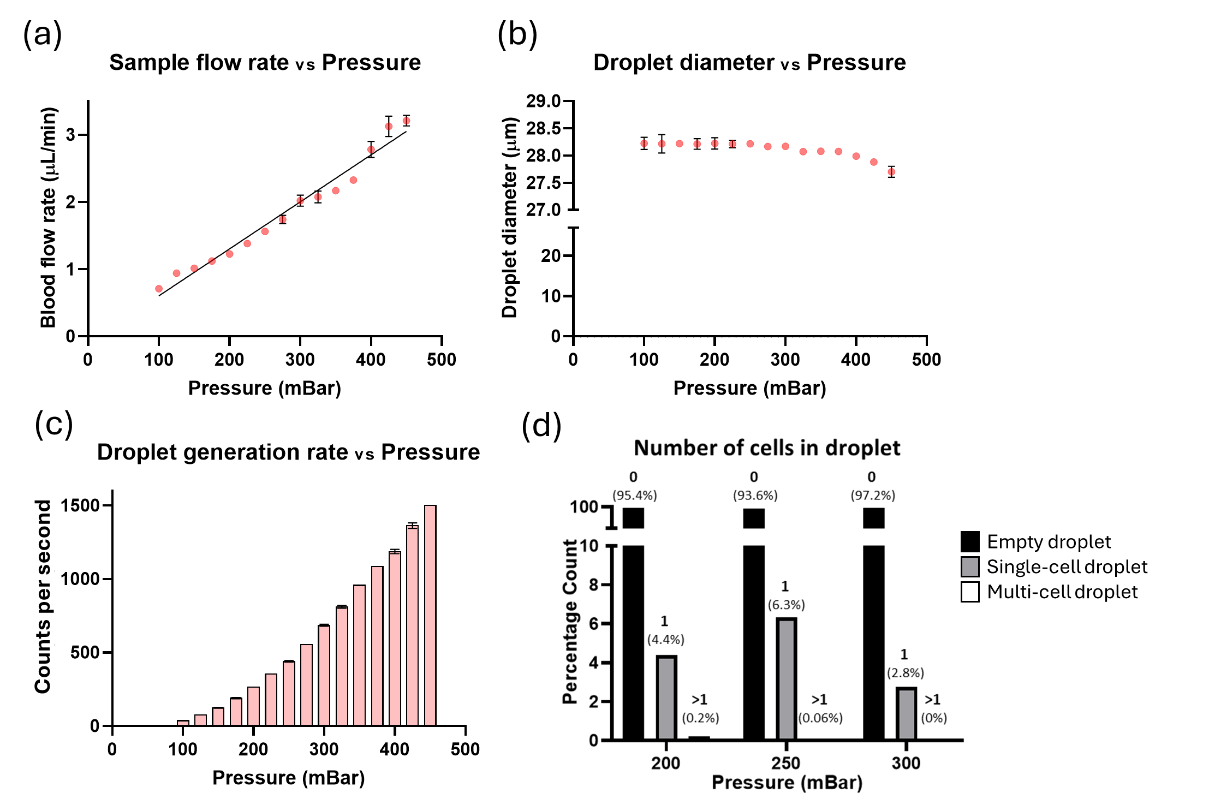


**Supplementary Figure S3.** Microfluidic device and experimental droplet generation characterisation figure reproduced from reference by Zeming et al[1]. (a) shows the driving pressure of the microfluidic device with respect to the sample flowrate. (b) characterizes the droplet size measurements based on the driving pressure while (c) shows the rate of droplet generation with respect to the driving pressure. Finally, the percentage of empty droplets, droplets with single cells and droplets with more than one cells with respect to pressure.


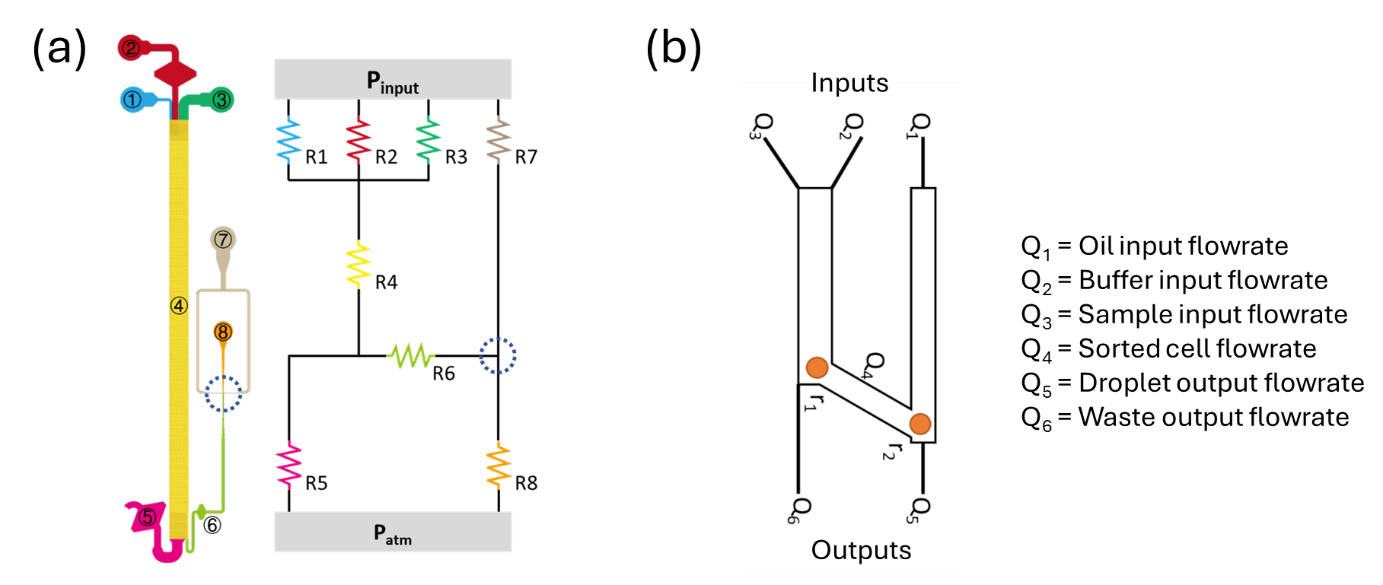


**Supplementary Figure S4.** Microfluidic designs and fluid flow schematics adapted from reference by Zeming et al[1]. (a) shows the microfluidic device design and resistivity circuit schematics for single cell droplet processing from blood samples. Blood samples are added to 2 shown in red. The resistivity for R_1_ + R_2_ + R_3_ is matched to R_4_ to ensure laminar and even distribution of flow widths into the DLD device in segment 4. The waste RBC is output to 5 while sorted immune cells are input into 6 to be encapsulated by the oil flow in 7. The final output with oil is indicated at 8. (b) shows the flow circuit to design the microfluidics by determining the ratio of flow to be distributed at r_1_ and r_2_. The calculations of flowrates determine the specifications of the device design. Briefly as described by Zeming et al. the flow rate equations result in the following equation where r_1_ is the ratio of fluid flow at the bifurcation Q_4_/Q_6_ and r_2_ describes Q_4_/Q_1_.

$r_{1}=\frac{1}{(1+\frac{1}{r_{2}})}$ ---- Supplementary Equation S1

Based on the desired flow specifications and same initial and outlet pressure, the microfluidic channel widths or bifurcations and incubation lengths can have the same resistance and tube distance. One set of meaningful r_1_ and r_2_ for design consideration of sorting and concentration of WBC is 1/4 and 1/3.


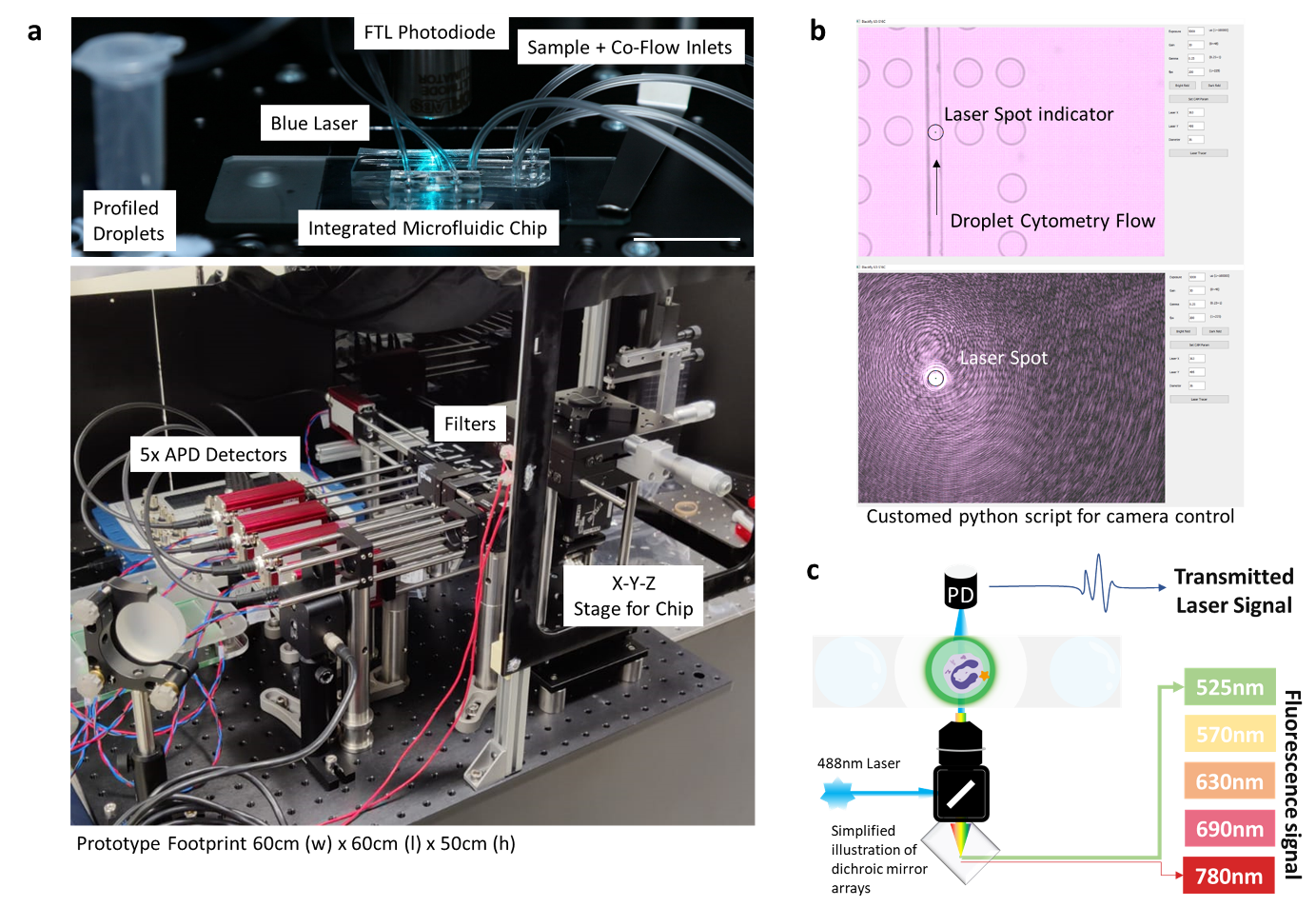


**Supplementary Figure S5.** Optical instruments of iSEAQ. Photos of iSEAQ microfluidic (top) and opto-electronic (bottom) components are shown in (a). Scale bar of the microfluidic chip shows 20 mm. (b) A screenshot of the customed python GUI for visualization to assist alignment of the droplet passage channel to the center of the collimated 488nm laser spot. (c) Illustration of the signal detection scheme from single-cell droplet. A Silicon photodiode (PD) with enhanced responsivity at UV-blue range is aligned with the microscope objective to detect the front transmitted laser (FTL) signal through the microfluidic chip. Cell-containing droplets were detected by fluctuations in FTL signal within the 2 boundary peaks.

**
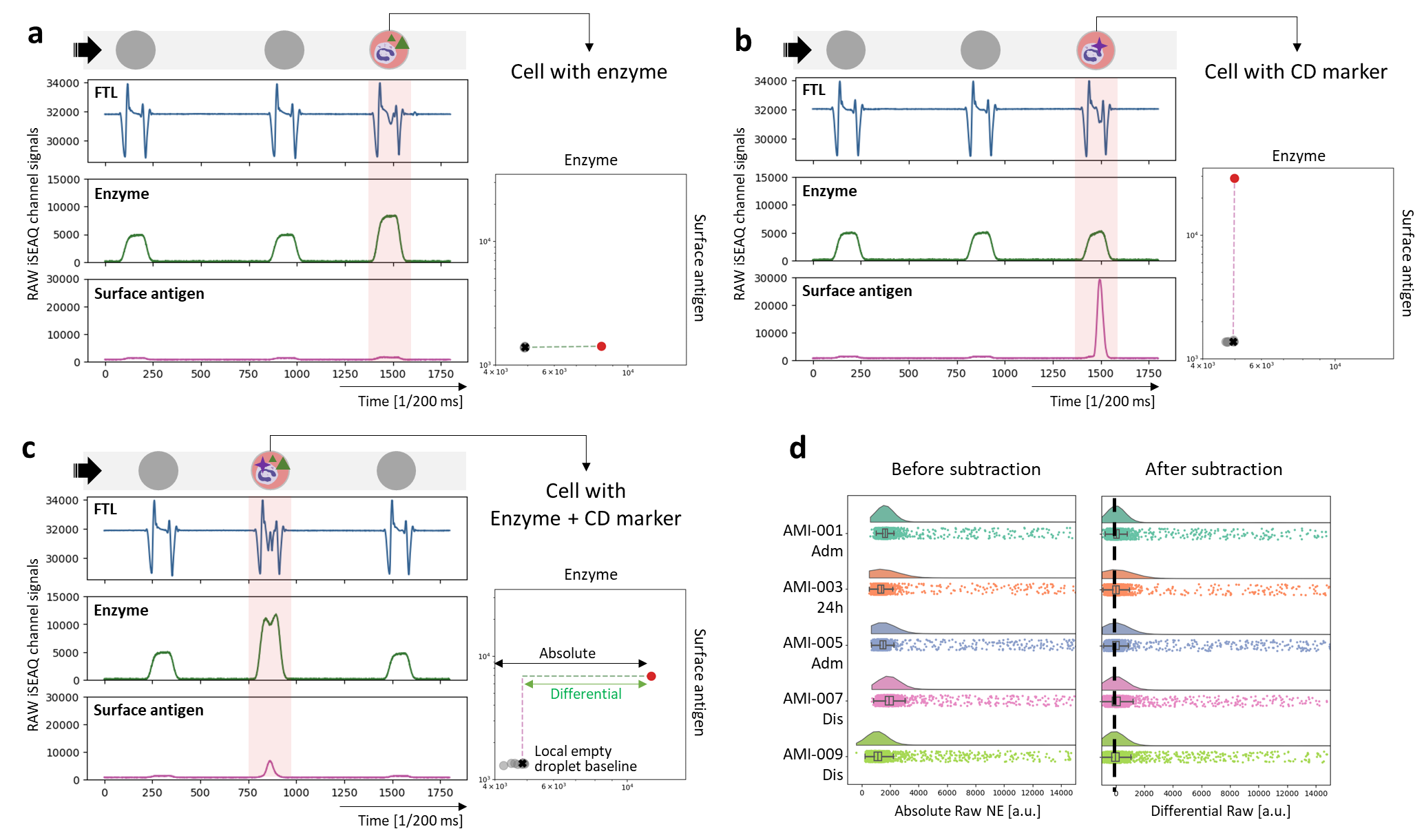
**

**Supplementary Figure S6.** Differential droplet signal measurement to minimize batch effect (a), (b), and (c), Real-time readings for a cell with enzyme secretion, a cell with CD marker expression and a cell with both enzyme secretion and surface antigen expression are shown in respectively. The scatter plots compare the readings of cell droplet (red) vs 10 nearest empty droplets (grey). The black cross labels the medians of enzyme and surface antigen readings. The dotted line corresponds to the Manhattan distance between the cell droplet reading and local empty droplet baseline, namely the differential raw reading in iSEAQ. (d) Readings of one iSEAQ channel (690nm) from 5 different donors conducted at various dates in the study are compared, before local background subtraction (absolute raw) and after local background subtraction. This shows that the differential raw can effectively correct the batch effect seen in the experiments.


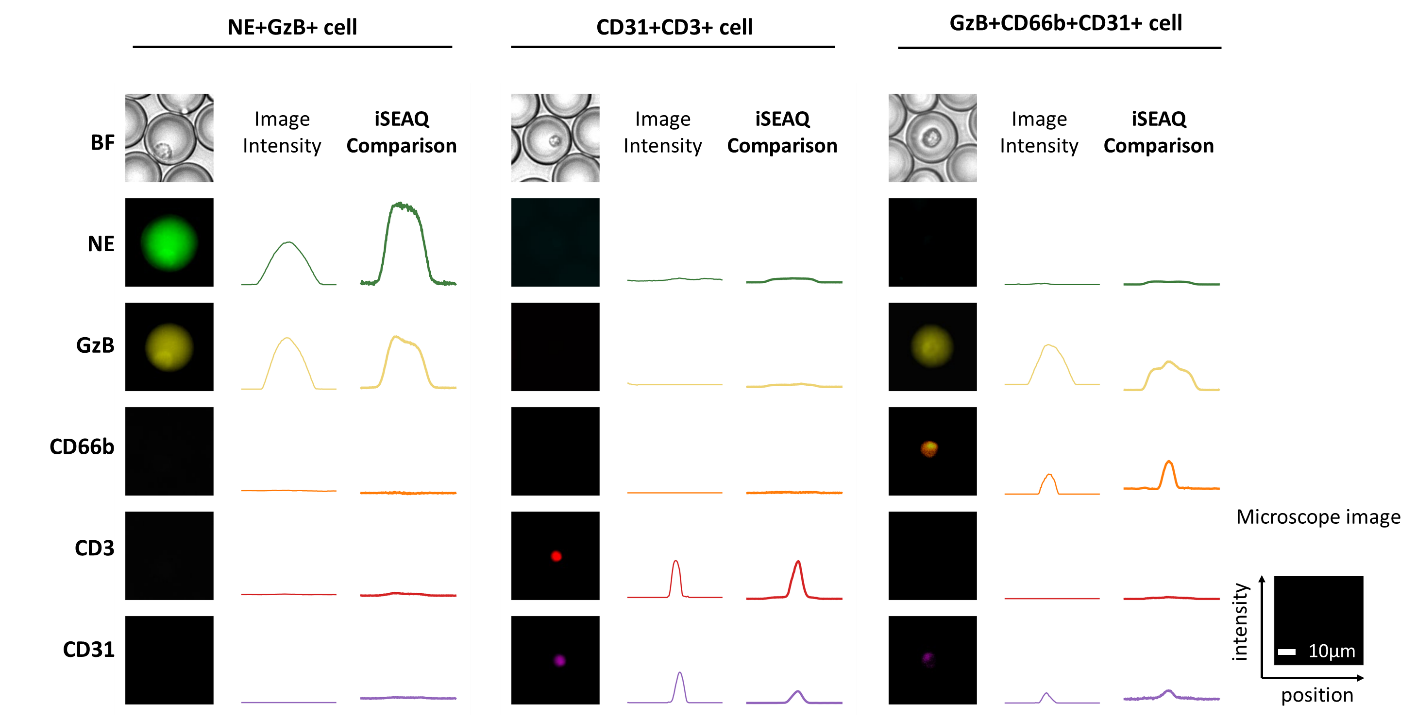


**Supplementary Figure S7.** Brightfield and fluorescence imaging of iSEAQ assay markers in selected droplet. Brightfield (BF) and fluorescence microscopy images show representative leukocytes with distinct combination of enzyme secretion (NE and GZB) and surface marker profile (CD66b, CD3 and CD31). Image taken using a monochrome camera and false color added to the image. The curves above each fluorescence image represent the scaled fluorescence signal of the droplet image in the longitudinal direction. Scale bar of images shown as 10 µm.


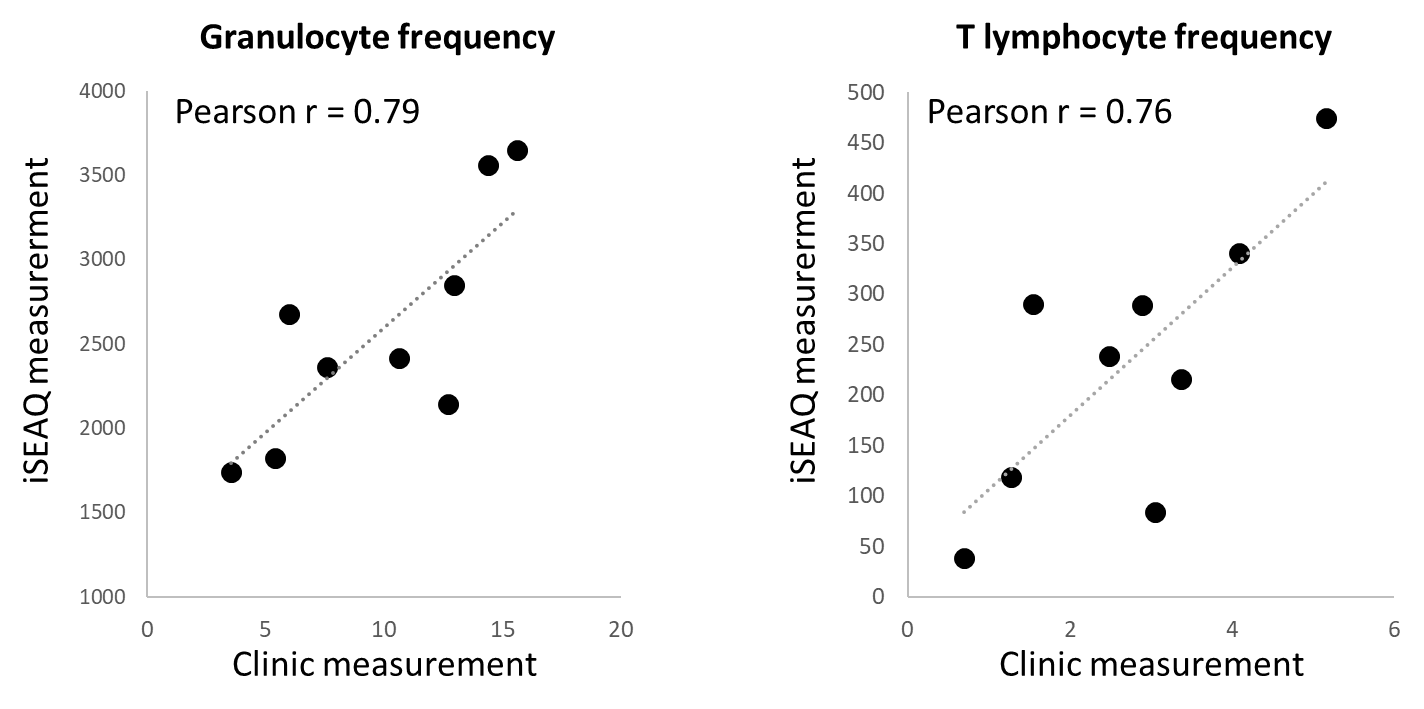


**Supplementary Figure S8.** Correlation between iSEAQ measurement frequency in counts per 100,000 droplets and clinic (flow cytometry) measured cell frequency (counts x10^9^ per Litre of blood). Pearsons correlation data is measured to be 0.79 and 0.76 for Granulocyte frequency versus CD66b+ and T lymphocyte frequency versus CD3+ respectively with two-tailed p value < 0.05.


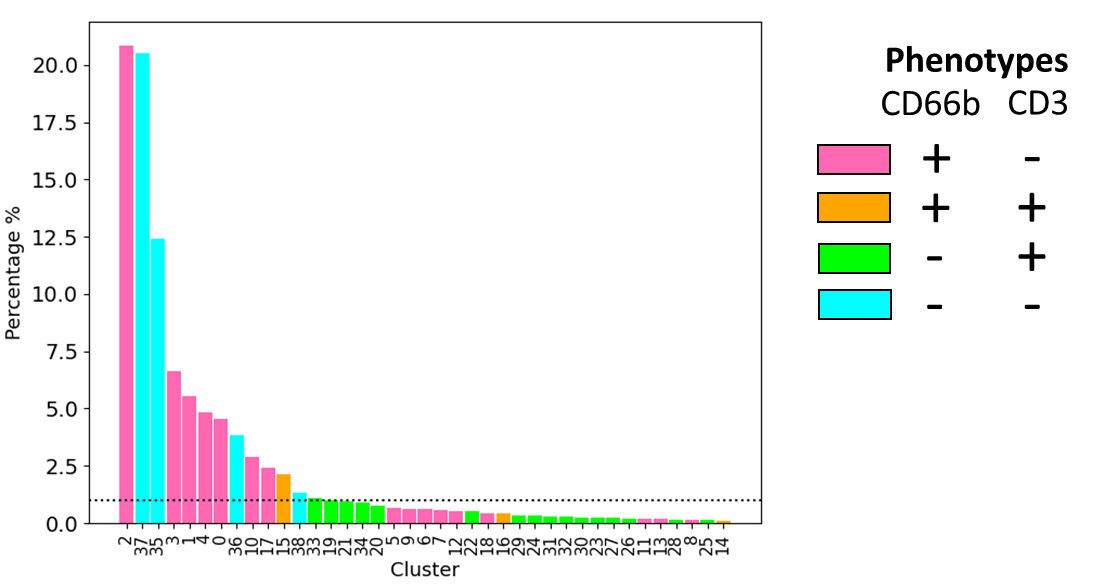


**Supplementary Figure S9.** Ranked UMAP clusters based on cluster size and cell counts. The dotted line indicates the 1% threshold for selected clusters for analysis where cluster 33 is the cut-off.


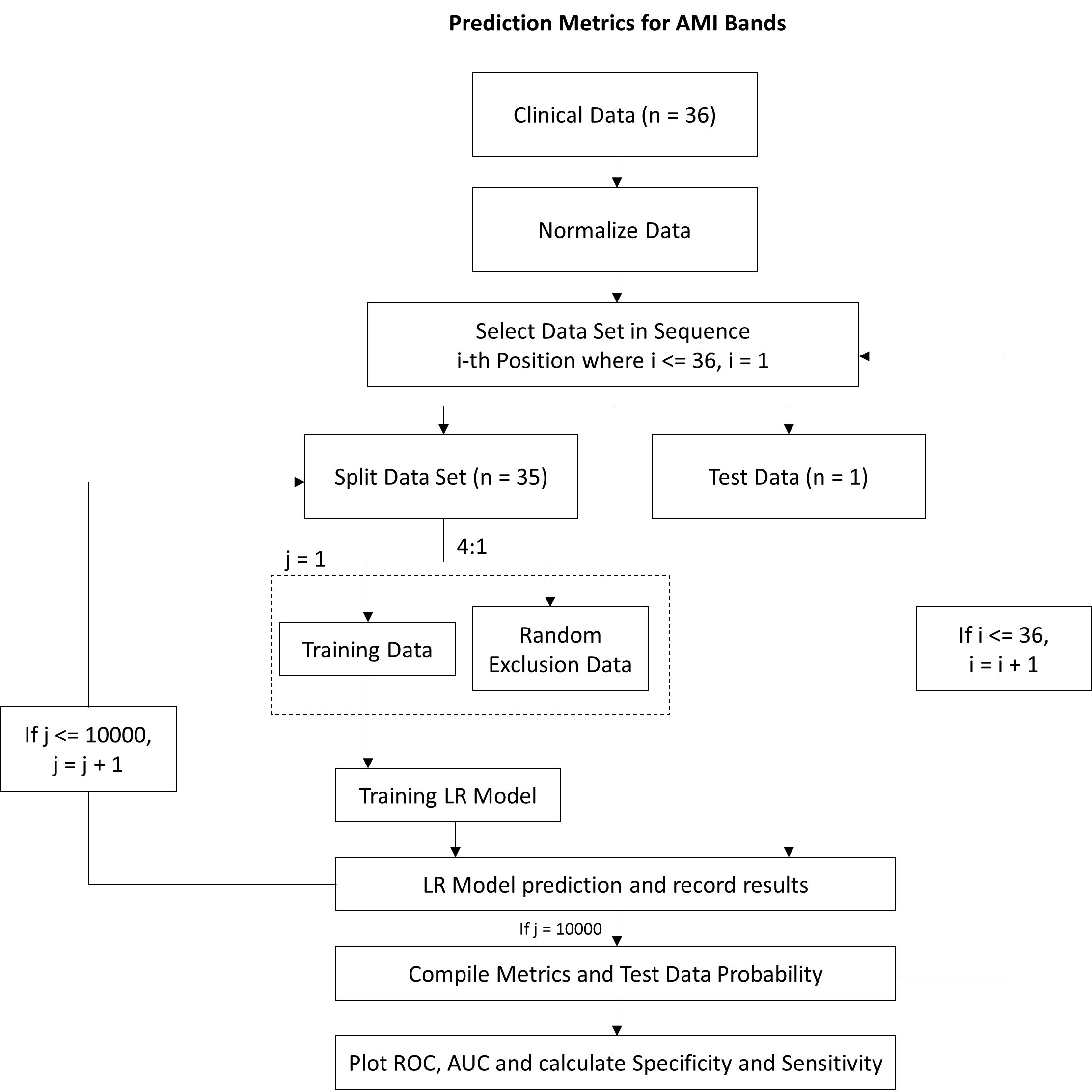


**Supplementary Figure S10.** Algorithm for the ROC plotting of iSEAQ 20 features for AMI banding and classification metrics calculation using the linear regression model.

Supplementary Discussion S1: Differential measurement of droplet signal

Supplementary Figure S3 illustrates the droplet signal readout for iSEAQ. Equations 3.1 to Equation 3.9 relate the raw channel readings [f_n_] from iSEAQ system to the actual fluorescence yield from the proteomic targets [F_enz_] (enzymes) and [F_CD_] (surface antigens). The first term in Equation 3.1 represents the contribution of fluorescence signal from all 5 fluorophores (5FAM, mfluor Blue 570, PE-dazzle, PE Cy5.5 and PE Cy7) to the raw reading of single channel, with the fluorescence spill-over attenuated by [c_kn_] coefficient for all except the primary fluorophore. The second term [ε] is the offset from 0V in voltage readings and it remains constant for all experiments. The spill-over coefficients are consistent for calibration and patient experiments and thus compensation matrix is applied across all experiment as the inversion of the spill-over coefficient matrix. The fluorophore is quenched initially for the NE substrate and the GzB.substrate. The initial quenched substrate concentration is denoted by [S]. After 30 minutes of droplet incubation, a portion of substrate can be cleaved by enzyme to yield a mixture of cleaved substrate [s] and remaining quenched substrate [S-s]. The fluorescence of quenched substrate can be estimated as a small fraction [$\epsilon$] of the fluorescence of cleaved substrate[6], as shown in Equation 3.2. The fluorescence yield from the enzyme channel in iSEAQ is essentially some constant K_enz_ times of the mixture of cleaved and quenched substrate (Equation 3.3 and 3.4), whereas the fluorescence from the surface antigen is directly K_CD_ times of the concentration. For simplicity for explanation, here we let K_enz_ and K_CD_ as 1 in Equation 3.7. Substituting [F_n_] in Equation 3.1 with the terms in Equation 3.5 and 3.6, the raw signal can be disintegrated into the sum of 5 discrete terms corresponding to 2 enzymes and 3 surface antigens. Equation 3.8 shows the breakdown of the empty droplet backgrounds, which only contains quenched substrate [S] and 0 surface antigen concentration. Equation 3.9 subtracts the cell-droplet signal in channel 1 [f_1_] with the empty droplet signal in the same channel [f_1,bg_],differential measurement of droplet signal in iSEAQ.

As shown in Equation 3.9, differential measurement theoretically eliminates the fluorescence contribution [S] by the quenched substrate. As Km/Kcat >> [S] are true for both NE and GzB substrates,[7, 8] differential measurement corrects for the experimental variation due to the preparation of the substrates. Moreover, differential measurement removes the offset [$\epsilon$] and other potential system variabilities and therefore it is taken as a robust parameter unique to iSEAQ, with direct correlation with enzyme activity and surface antigen expression.


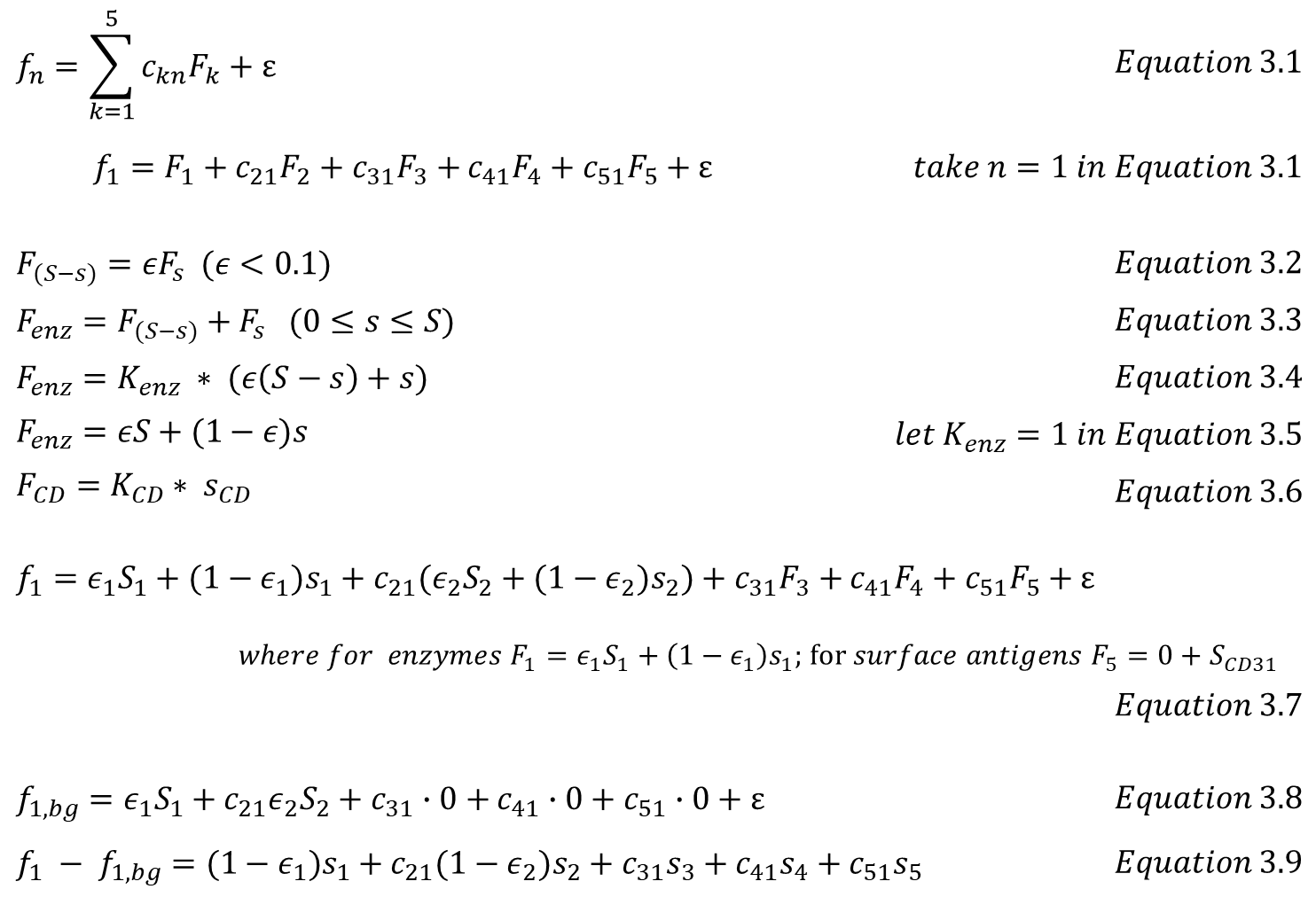


References

1. Zeming, K.K., et al., *Multiplexed Single-Cell Leukocyte Enzymatic Secretion Profiling from Whole Blood Reveals Patient-Specific Immune Signature.* Analytical Chemistry, 2021. **93**(10): p. 4374-4382.

2. Yoon, J., A. Terada, and H. Kita, *CD66b Regulates Adhesion and Activation of Human Eosinophils.* The Journal of Immunology, 2007. **179**(12): p. 8454-8462.

3. Puellmann, K., et al., *A variable immunoreceptor in a subpopulation of human neutrophils.* Proc Natl Acad Sci U S A, 2006. **103**(39): p. 14441-6.

4. Bagwell, C.B., et al., *Multi-site reproducibility of a human immunophenotyping assay in whole blood and peripheral blood mononuclear cells preparations using CyTOF technology coupled with Maxpar Pathsetter, an automated data analysis system.* Cytometry Part B: Clinical Cytometry, 2020. **98**(2): p. 146-160.

5. Davis, J.A., et al., *Deterministic hydrodynamics: taking blood apart.* Proceedings of the National Academy of Sciences of the United States of America, 2006. **103**(40): p. 14779-84.

6. Clegg, R.M., *Chapter 1 Förster resonance energy transfer—FRET what is it, why do it, and how it's done*, in *Laboratory Techniques in Biochemistry and Molecular Biology*. 2009, Elsevier. p. 1-57.

7. Schulenburg, C., et al., *A FRET-based biosensor for the detection of neutrophil elastase.* Analyst, 2016. **141**(5): p. 1645-8.

8. Kula, T., et al., *T-Scan: A Genome-wide Method for the Systematic Discovery of T Cell Epitopes.* Cell, 2019. **178**(4): p. 1016-1028.e13.
